# Dose-Dependent Physiological and Transcriptomic Responses of Lettuce (*Lactuca sativa* L.) to Copper Oxide Nanoparticles—Insights into the Phytotoxicity Mechanisms

**DOI:** 10.3390/ijms22073688

**Published:** 2021-04-01

**Authors:** Tiantian Xiong, Shasha Zhang, Zhuangzhuang Kang, Ting Zhang, Shaoshan Li

**Affiliations:** Key Laboratory of Ecology and Environmental Science in Guangdong Higher Education, School of Life Science, South China Normal University, Guangzhou 510631, China; xiongtiantian@m.scnu.edu.cn (T.X.); 2018022513@m.scnu.edu.cn (S.Z.); kangzhzh@m.scnu.edu.cn (Z.K.); ting.zhang2@m.scnu.edu.cn (T.Z.)

**Keywords:** CuO-NPs, foliar exposure, transcriptomics, photosynthesis, oxidative stress

## Abstract

Understanding the complex mechanisms involved in plant response to nanoparticles (NPs) is indispensable in assessing the environmental impact of nano-pollutants. Plant leaves can directly intercept or absorb NPs deposited on their surface; however, the toxicity mechanisms of NPs to plant leaves are unclear. In this study, lettuce leaves were exposed to copper oxide nanoparticles (CuO-NPs, 0, 100, and 1000 mg/L) for 15 days, then physiological tests and transcriptomic analyses were conducted to evaluate the negative impacts of CuO-NPs. Both physiological and transcriptomic results demonstrated that CuO-NPs adversely affected plant growth, photosynthesis, and enhanced reactive oxygen species (ROS) accumulation and antioxidant system activity. The comparative transcriptome analysis showed that 2270 and 4264 genes were differentially expressed upon exposure to 100 and 1000 mg/L CuO-NPs. Gene expression analysis suggested the ATP-binding cassette (ABC) transporter family, heavy metal-associated isoprenylated plant proteins (HIPPs), endocytosis, and other metal ion binding proteins or channels play significant roles in CuO-NP accumulation by plant leaves. Furthermore, the variation in antioxidant enzyme transcript levels (*POD1, MDAR4, APX2, FSDs*), flavonoid content, cell wall structure and components, and hormone (auxin) could be essential in regulating CuO-NPs-induced stress. These findings could help understand the toxicity mechanisms of metal NPs on crops, especially NPs resulting from foliar exposure.

## 1. Introduction

Metal nanoparticles are a new class of pollutants with the features of both metals and nanoparticles (NPs). These peculiar materials have raised global concerns about the possible environmental toxicity due to their structure and the unique physical and chemical properties relative to the bulk metals [1]. Copper oxide nanoparticles (CuO-NPs) are widely used in solar cells, gas sensors, catalysis, electronics, cosmetics, fuel additives, and textile industries. Furthermore, CuO-NPs have many applications in the healthcare, paint, plastics, and agricultural sectors (i.e., nano pesticides, fungicides, and wood preservatives). However, their increasing use in agro-ecological systems has attracted scientific attention due to their potential toxicity [2,3,4,5].

Plants are considered the most significant interface between the biosphere and the environment; they directly interact with the air, soil, and water [6]. Research has reported the uptake and translocation of NPs from the roots to the leaves and adverse plant responses to NPs. Also, engineered NPs released into the environment could reach the plant leaves [7,8], especially when used in agriculture for plant protection. For example, plant leaves are usually sprayed with NPs to resist to phytopathogenic fungus on vines [9,10,11]. According to Keller et al. (2018), lettuce can retain a significant amount of CuO-NPs on the leaf surface, which was taken up by the leaves, resulting in human exposure [12]. Foliar uptake is an important pathway by which leafy vegetables intercept and accumulate NPs. However, the fate and phytotoxicity mechanisms of NPs after leaf exposure remain unclear.

The effect of NPs in higher plants have been reported in numerous recent studies, including both positive [13,14] and adverse effects [8,15]. According to Gkanatsiou et al. (2019), Cu-based NPs (Cu NPs: 100, 150, and 200 μg/mL with hydrodynamic size of 172 ± 12 nm and Cu_2_O NPs: 150, 200, and 250 μg/mL with hydrodynamic size of 197 ± 8 nm) can penetrate plant leaves without causing any toxicity; no significant differences were observed in chlorophyll content, quantum yield, CO_2_ assimilation, and plant growth in pot experiments (in vivo) [16]. Adhikari et al. (2016) revealed that 8 ppm foliar spray and 0.02 ppm root exposure of CuO-NPs could easily enter plant cells and regulate different enzyme activities to enhance plant growth [17].

However, other studies have demonstrated that CuO-NPs at different concentrations adversely affect the growth of lettuce, radish, cucumber, rice, alfalfa, maize, and barley, in a dosage-dependent manner [15,17,18,19,20,21,22]. According to Hong et al. (2015b), the exposure of cucumber seedling leaves to engineered CuO-NPs (at 50, 100, and 200 mg/L) induces phytotoxic symptoms, observed as a decrease in transpiration and net photosynthesis rates [23]. In the study of Atha et al. (2012), a strong plant growth inhibition were observed for radish (*Raphanus sativus*), perennial ryegrass (*Lolium perenne*), and annual ryegrass (*Lolium rigidum*) after treated with CuO-NP and bulk CuO suspensions at 10, 100, 500, and 1000 mg/L [24]. In rice (*Oryza sativa*, var. Jyoti), CuO-NPs significantly decreased the germination rate, root and shoot length, and biomass at high concentrations (100 or 1000 mg/L) [25]. Overall, the effects of Cu-based NPs on plants appear at different exposure levels and conditions, depending on the NP characteristics and plant species [26].

Recent transcriptome analysis has provided new insights into the molecular mechanisms of NP toxicity on plants [27]. Although the study revealed the influence of NPs on gene expression, the transcriptome-wide molecular mechanism underlying the effect of different concentrations of CuO-NPs is still unclear. Chen et al. (2019) reported that low concentrations of CuO NPs (1 mg/L) could not induce significant gene expression changes in *Desulfovibrio vulgaris*, while 50 and 250 mg/L caused substantial transcriptional changes [28]. Evaluation of the transcription responses enriched in the cellular processes of motility and ion transport, electron transfer, and energy production.

In the present study, CuO-NPs were sprayed on lettuce (*Lactuca sativa* L.) leaf surfaces at different concentrations to explore the biological effects and mechanisms of Cu-based NPs. Subsequently, the physiological and transcriptomic responses of the plants were studied. This study may provide information regarding the impact of atmospheric deposition of CuO-NPs on above-ground plant tissues and the safety of using CuO-NPs in agricultural systems.

## 2. Results and Discussion

### 2.1. Biomass and Leaf Area

**Figure 1:**
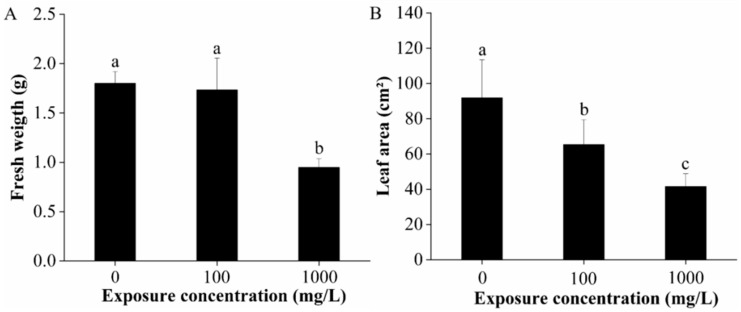
Fresh weight (**A**) and leaf area (**B**) of lettuce after 15 days of foliar exposure to CuO-NPs (0, 100, and 1000 mg/L). Values are expressed as the mean of five replicates (± SD) for each treatment; the different lowercase letters (a, b, and c) indicate significant difference at *p* < 0.05 among different exposure concentrations, mean values with the same letter are not significantly different.

CuO-NPs had no significant influence on the fresh weight of lettuce at 100 mg/L, but significantly decreased the fresh weight by 47.3% at the concentration of 1000 mg/L (*p* < 0.01) (Figure 1A). Meanwhile, the leaf areas were significantly reduced by 28.8% and 54.8% after 100 and 1000 mg/L CuO-NPs treatment, respectively (Figure 1B). The results show that CuO-NPs significantly inhibited lettuce growth after 15 days of treatment and these effects were dose-dependent.

Several recent studies have also reported the toxicity effect of CuO-NPs after foliar spay [8,15,29]. Hong et al. (2015b) reported that foliar application with CuO-NPs (50, 200 mg/L) significantly reduced cucumber firmness, compared with control [23]. Xiong et al. (2017) observed the decrease of lettuce weight, water content and necrosis on leaf surface after foliar application with 10 and 250 mg CuO-NPs per plant [8]. According to the study of Yue et al. (2018), CuO NPs (150μg/L, exposure for 7 days) significantly decreased the dry weights, frond number, and frond surface area of duckweed (*Lemna minor*. L) by 32%, 47%, and 33%; and the responses were dose-dependent [30].

The study of the impacts of foliar exposure of NPs on plant growth is rather limited, but is important for comprehensive understanding the effect of NPs both on the underground and above-ground plant portions.

### 2.2. Gas Exchange, Photosynthetic Pigments Concentration, and Chlorophyll Fluorescence

**Figure 2:**
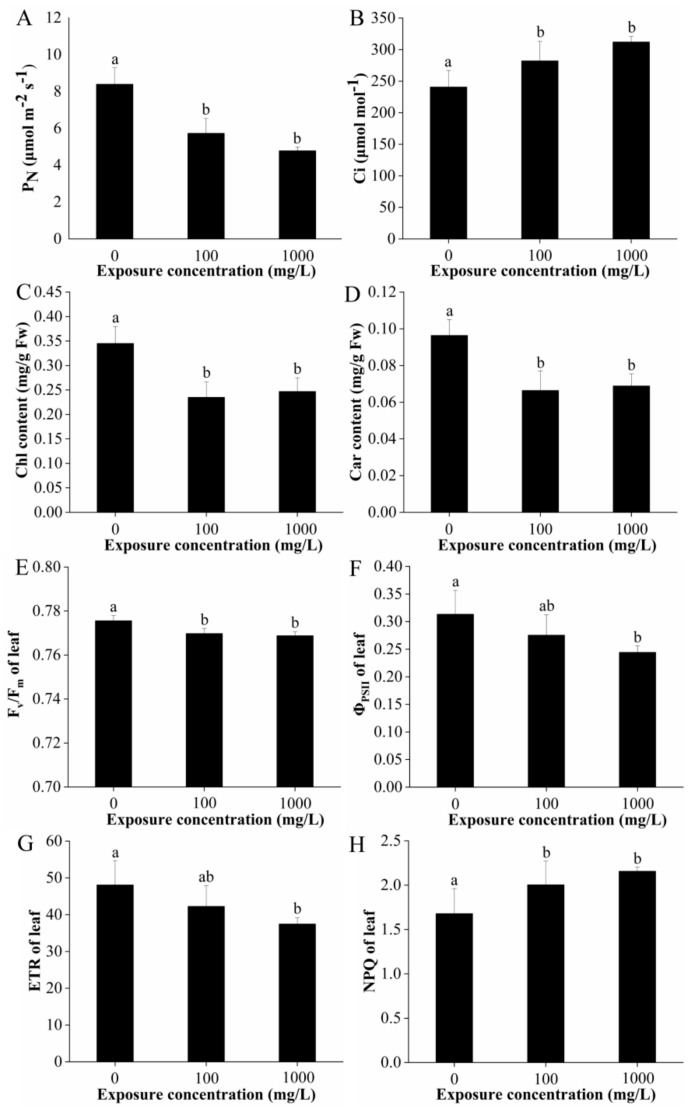
Gas exchange parameters (net photosynthesis (**A**), intercellular carbon dioxide concentration (**B**), total chlorophyll (**C**), and carotenoids (**D**) content, and chlorophyll fluorescence parameters (potential efficiency of PSII photochemistry (F_v_/F_m_, **E**), effective quantum yield of PSII (Φ_PSII_, **F**), electron transport rate (ETR, **G**), and non-photochemical fluorescence quenching (NPQ, **H**)) of lettuce after 15 days of foliar exposure to CuO-NPs (0, 100, and 1000 mg/L). Values are expressed as the mean of five replicates (± SD) for each treatment; the different lowercase letters (a and b) indicate significant difference at *p* < 0.05 among different exposure concentrations, mean values with the same letter are not significantly different.

The light saturation point of lettuce leaves is between 1200–1500 µmol/m^2^·s; thus, a light intensity of 1500 μmol m^−2^ s^−1^ was chosen to determine the net photosynthesis (P_N_), stomatal conductance (g_s_), intercellular carbon dioxide concentration (C_i_), and transpiration rate (T_r_) of each sample. The results showed that the P_N_ of lettuce treated with 100 and 1000 mg/L CuO-NPs was decreased by 31.7% and 43.0% significantly, respectively, while the C_i_ was significantly increased by 17.2% and 29.6%, respectively (Figure 2A,B). However, there were no significant differences in g_s_ and T_r_ after exposure to 100 and 1000 mg/L CuO-NPs (data not presented).

Chlorophylls are the core photosynthetic pigments that absorb light in the chloroplast and transduce it into chemical energy [31]. The content of chlorophyll a (Chl a), chlorophyll b (Chl b), and total chlorophyll (Chl) were dropped by 31.9% and 28.9%, 31.7% and 25.7%, 31.9%, and 28.4% following treatment with 100 and 1000 mg/L CuO-NPs, respectively (Figure S1, Figure 2C). The carotenoid (Car) content was also reduced by 31.7% and 25.7% in the 100 and 1000 mg/L CuO-NPs treatment groups, respectively, relative to the control (Figure 2D).

The fluorescence parameters are presented in Figure 2E–H, including the potential efficiency of photosystem (PS) II photochemistry (F_v_/F_m_), the effective quantum yield of PSII (Φ_PSII_), the electron transport rate (ETR), and the non-photochemical fluorescence quenching (NPQ). In the present study, exposure to 100 and 1000 mg/L CuO-NPs significantly reduced the F_v_/F_m_ of lettuce leaves by 0.75% and 0.88%. No significant effects on Φ_PSII_ and ETR were observed in the 100 mg/L CuO-NPs treatment group, but these values significantly decreased by 22.0% (both Φ_PSII_ and ETR) in the 1000 mg/L CuO-NPs group, respectively. In contrast, NPQ was significantly increased by 19.3% and 28.4% in the 100 and 1000 mg/L CuO-NPs treatment groups.

The results show that CuO-NPs at both 100 and 1000 mg/L greatly reduced the net photosynthesis and chlorophyll and carotenoids content in lettuce. Photosynthesis is the basic function determining productivity of green plants [32]. In the study of Xiong et al. (2017), the photosynthetic activity (net photosynthesis and stomatal conductance) significantly decreased after foliar application of CuO-NPs (0, 10, and 250 mg per plant) [8]. In the study of Costa et al. (2016), the photosynthetic rate and pigment contents declined with a complete loss of PSII photochemical quenching at 1000 mg/L CuO-NP [25]. Nekrasova et al. (2011) reported that low Cu-based NPs concentrations (0.025, 0.25, and 0.5 mg/L) has a positive impact on photosynthesis of *Elodea densa*, while a significant inhibitory effect was observed when concentrations are higher than 1 mg/L [32]. In the present study, the photosynthetic rate was significantly affected by the increasing CuO-NPs concentrations. This can be attributed directly to oxidative stress [33] or to inactivation of ribulose biphosphate (RuBP) carboxylase, a key enzyme in photosynthetic CO_2_ fixation, due to copper interaction with SH groups [32]. The accumulation of Cu inside cells in roots and leaves, specifically the latter, is associated mainly with structural damages such as deformation of stomata and chloroplasts, with a low number of thylakoids per grana in *Oryza sativa*, *Lactuca sativa*, and *Brassica oleracea* var capitata [8,25,34], this may further decrease light-harvesting and result in an inhibition in photosynthesis [8].

Additionally, the reduction of pigment contents in plant leaves could also influence photosynthetic rate [32]. Amounts of studies have demonstrated that the application of CuO-NP depressed the concentration of photosynthetic pigments (chlorophyll a and b and carotenoids) in terrestrial (*Triticum aestivum*, *Vigna radiata* L., *Oryza sativa*) and aquatic plants *(Elodea densa, Landoltia punctate, Lyngbya majuscula*) [25,32,35,36,37]. Several studies also found that, after sprayed with Cu-NPs at doses of 100, 150, and 200 μg mL^−1^ and Cu_2_O-NPs at doses of 150, 200, and 250 μg mL^−1^ on plant leaves, both Cu and Cu_2_O NPs did not cause any chlorophyll content reduction in bean plants (*P*. vulgaris) [16]. In cilantro, Cu-based NPs did not affect chlorophyll production [26]. The evidence from a diverse array of studies demonstrate that chlorophyll concentration increases in response to low-level stress and decreases in response to high-level stress [31]. According to the previous study, the chlorophyll activity may be influenced both by the mechanical coating of NPs on leaf surface or by the accumulated Cu within plant [34,38,39]. Moreover, chloroplasts is one of the sources of ROS generation, the reduction of chlorophyll in plants upon exposure to CuO-NPs may attributed directly to oxidative stress [40]. Carotenoids are known to be light harvesting pigments by absorbing photons and transferring the excitation energy to chlorophyll, which eventually reaches the reaction center, they also act as potent quenchers of ROS, particularly singlet oxygen (^1^O_2_) by intercepting the triplet-chlorophyll [20]. Decreased chlorophyll and carotenoids levels in the present study might render the CuO-NP exposed lettuce more vulnerable to stress.

Moreover, the chlorophyll fluorescence measurements in the present study revealed an impairment of the primary photochemical reactions of photosynthesis in both CuO-NPs treatment groups. Published studies have shown that high amounts of Cu decrease photosynthesis due to altered photochemical reactions in photosystem II (PSII) and damage to plant growth [41]. Consistent with our study, Perreault et al. (2010) found that CuO-NPs affected chlorophyll fluorescence in *Lemna gibba*, causing a decrease in the PSII maximal, PSII operational quantum yields, electron transport capacity of PSII and an increase in thermal energy dissipation [42]. The reduction in Φ_PSII_ and ETR characterizes a decrease in the photochemical efficiency of PS II, resulting in excess electron accumulation, ROS production, and subsequent photodamage to the plant [43,44]. Plants cope with excess electron generation by photochemical fluorescence quenching (qN) and/or NPQ through heat dissipation [44]. The increase in NPQ we observed after CuO-NP treatment indicates that lettuce could use this mechanism to manage excess energy generation.

In summary, the photosynthetic rate, photosynthetic pigments, and chlorophyll fluorescence parameters of lettuce were significantly affected upon exposure to 100 and 1000 mg/L CuO-NPs. The impairment on plant photosynthesis probably results from the oxidative stress, the structural and functional change of the photosynthetic apparatus, and the imbalance in the pigment complex. Additionally, the reduction in leaf area (shown in Figure 1B) could limit the surface area for photosynthetic rate and water uptake, and consequently affects the plant performance.

### 2.3. Reactive Oxygen Species (ROS) Determination and Antioxidant Activities in Plant Leaves

**Figure 3:**
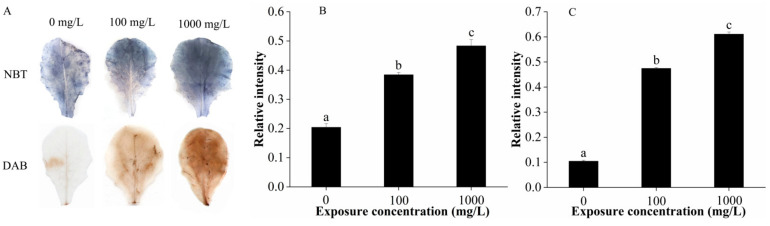
Generation of O_2_^−^ and H_2_O_2_ determined by NBT and DAB dye (**A**); the corresponding quantitative results of O_2_^−^ (**B**) and H_2_O_2_ (**C**) generation in lettuce leaves after 15 days of foliar exposure to CuO-NPs (0, 100, and 1000 mg/L). Values are expressed as the mean of three replicates (± SD) for each treatment; the different lowercase letters (a, b, and c) indicate significant difference at *p* < 0.05 among different exposure concentrations.

In Figure 3A, nitroblue tetrazolium (NBT) staining of CuO-NPs stressed leaves exhibited dark blue spots (insoluble formazan), indicating the generation of superoxide anion (O_2_^−^). In the 1000 mg/L CuO-NPs treatment group, the blue areas formed by O_2_^−^ covered the entire leaf surface. Meanwhile, 3′3′-diaminobenzidine (DAB) staining of stressed leaves revealed deep brown spots, which pinpoints hydrogen peroxide (H_2_O_2_) generation. The number of spots increased with an increase in CuO-NPs concentration. Dark blue and deep brown spots indicated ROS accumulation/localization and severe oxidative burst under CuO-NP stress. Corresponding quantitative O_2_^−^ and H_2_O_2_ measurements are shown in Figure 3B,C. The relative intensities increased with exposure concentration, which are consistent with the observed NBT and DAB dyeing results.

**Figure 4:**
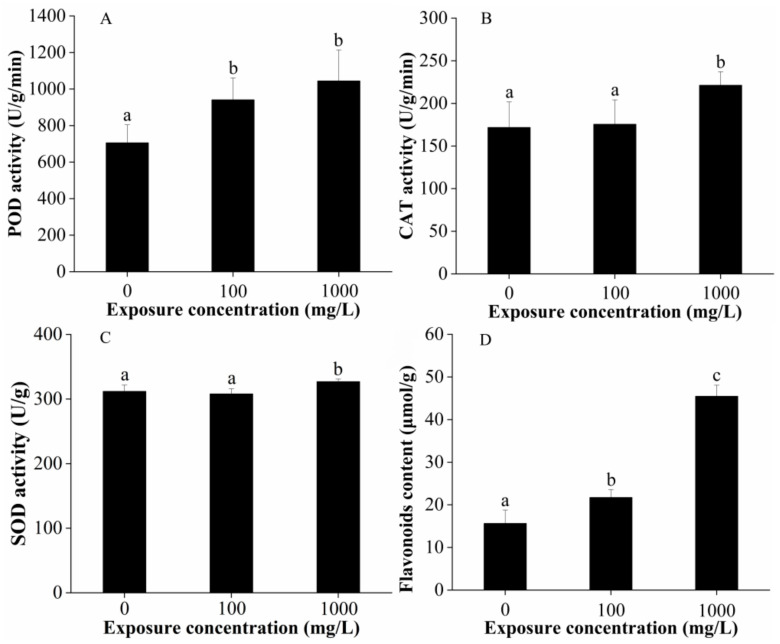
POD activity (**A**), CAT activity (**B**), SOD activity (**C**) and flavonoids content (**D**) in lettuce leaves after 15 days of foliar exposure to CuO-NPs (0, 100, and 1000 mg/L). Values are expressed as the mean of five replicates (± SD) for each treatment; the different lowercase letters (a, b, and c) indicate significant difference at *p* < 0.05 among different exposure concentrations, mean values with the same letter are not significantly different.

Moreover, CuO-NP exposure modulated the antioxidant enzyme activities of lettuce leaves. The peroxidase (POD; EC 1.11.1.7) activity increased significantly by 37.2% and 49.6% following treatment with 100 and 1000 mg/L CuO-NPs (Figure 4A). The catalase (CAT; EC 1.11.1.6) activity did not change after treatment with 100 mg/L CuO-NPs but was significantly increased (by 28.8%) when treated with 1000 mg/L CuO-NPs (Figure 4B). Similar to CAT activity, no significant effects on the superoxide dismutase (SOD; EC 1.15.1.1) activity were observed in the 100 mg/L CuO-NPs treatment group, but these values significantly increased by 4.80% in the 1000 mg/L CuO-NPs group (Figure 4C). This result could suggest that the POD is more sensitive to CuO-NPs treatment than CAT and SOD activity. Flavonoids occur widely in plants and form an interface between plants and the environment; their role as signals has been intensively studied [45,46]. In this study, the flavonoid (an antioxidant) contents of lettuce leaves were remarkably increased by 38.9% and 190.7% following treatment with 100 and 1000 mg/L CuO-NPs (Figure 4D).

The O_2_^−^ and H_2_O_2_ are considered primary ROS. The generation O_2_^−^ is mainly associated with electron transport chains, thus the major sources of O_2_^−^ within plant cells are chloroplast and mitochondria in complexes I and III, and PSI and PSII, respectively [47,48]. The H_2_O_2_ can impose a more severe oxidative stress condition due to its higher stability when compared to O_2_^−^ [48]. Our results show that CuO-NPs at both 100 and 1000 mg/L significantly induced oxidative stress to lettuce, as indicated by the increased generation of O_2_^−^ and H_2_O_2_.

Plants have several antioxidative defense systems to scavenge toxic radicals, in order to protect themselves from the oxidative stress. This defensibility is divided into two main classes: non-enzymatic antioxidants, like glutathione (GSH), ascorbic acid (AsA), phenolic compounds and proline (Pro), flavonoids, and carotenoids; and antioxidative enzymes, which comprise SOD, ascorbate peroxidase (APX), CAT, and glutathione reductase (GR) [47,49]. Among these antioxidant, peroxidases are involved in the intracellular detoxification of H_2_O_2_ by the oxidation of distinct chemical substrates [50]. Catalase is a tetrameric heme-containing protein, which is responsible for the intracellular detoxification of H_2_O_2_ by dismutating it into H_2_O and O_2_ [47]. SOD is a typical enzyme with significant antioxidant potency [49], as its activity directly modulates the amount of O_2_^−^ and H_2_O_2_. Flavonoids are wildly distributed secondary metabolites, they can direct interaction with ROS (e.g., singlet oxygen (^1^O_2_)and H_2_O_2_) and are able to serve as substrate for different peroxidases [45,46]. In the present study, the increments in POD, CAT, and SOD activity and flavonoid content in lettuce leaves as a result of foliar application of CuO-NPs suggest the induction of antioxidants that protect lettuce against ROS. Similar results were also found by Jonapá-Hernández et al. (2020), the foliar application of Cu-NPs (250, 500, 750, and 1000 ppm) for 120 h caused significantly changes in flavonoids, anthocyanins, phenylalanine ammonia lyase, and POD activities in *Annona muricata* L. leaves [51]. The application of Se and Cu nanoparticles (20 and 50 mg/L) on tomato leaves generated an induction of the activity of the enzymes SOD, ascorbate peroxidase, glutathione peroxidase, and phenylalanine ammonialyase in the leaves, and the enzyme glutathione peroxidase in the fruit [52]. The root extracts from wheat grown with the CuO NPs had higher POD and CAT activities than the extracts from the control plants [53]. In *Elodea densa*, SOD activity was 1.5–2.5 higher than in the control at any copper concentration (0.025, 0.25, 0.5, 1, and 5 mg/L CuO-NP) [32]. The activities of SOD and CAT are stimulated in CuO-NP (10, 50, 100, and 500 mg/L) treated tomato and cauliflower plant [40].

The study of Ogunkunle et al. (2018) suggested that the increased tolerance of plants to metal stress is linked to their ability to maintain efficient antioxidant systems in order to scavenge or detoxify excess toxic ROS in cells and tissues [54]. In addition, the accumulation capacities of different antioxidant enzymes depend on plant species [10]. In the present study, the synthesis rates of the antioxidant were markedly induced by CuO-NPs to scavenge excess ROS, alleviating phytotoxicity to the stressed lettuce.

### 2.4. Overview of Transcriptome Sequencing Analysis

To study the effects of CuO-NPs on lettuce after foliar exposure at the transcriptome level, lettuce leaves (15 days) were subjected to RNA extraction and transcriptome sequencing analysis. Three biological replicates for the 0 (CK), 100 mg/L (T1), and 1000 mg/L (T2) were used to ensure statistical comparability and the reliability of the data. The raw data ranged from 49,078,068 to 84,694,460 reads per sample (Table S1). More than 48 million clean reads were generated after remove low-quality reads, adaptor sequences, poly A and known non-coding RNAs (Table 1 and Table S1). The guanine-cytosine percentage (GC%) of sequenced data from nine samples ranged from 45.42% to 47.32%, and the percentage of reads with an average quality score >30 was about 93.78% (Table 1), indicating that the accuracy and quality of the sequencing data are sufficient for further analysis. The mapping efficiency of nine samples to the lettuce (*Lactuca sativa*) genome range from 89.5% to 94.9%, and the unique mapping rates were ranging from 81.6% to 90.5%, respectively (Table 1). A total of 24,585 unigenes were annotated.

**Figure 5:**
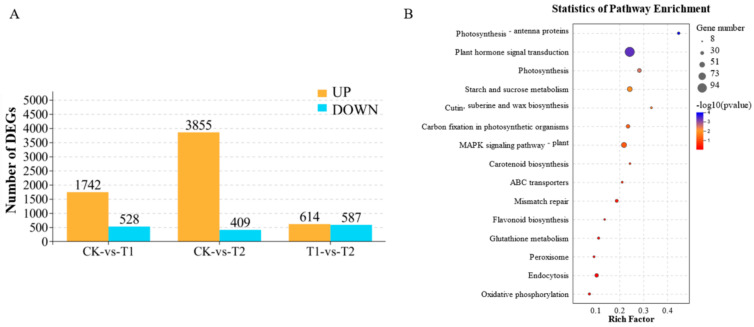
DEGs between different treatment groups (CK−vs−T1, CK-vs-T2, and T1-vs-T2) after 15 days of foliar exposure to CuO-NPs (0 (CK), 100 (T1), and 1000 (T2) mg/L) (**A**). Three replicates were prepared for each treatment. The genes/transcripts with a false discovery rate (FDR) below 0.05 and absolute fold change ≥2 were considered differentially expressed. Functional enrichment of the DEGs following treatment with CuO-NPs (**B**). Rich factor is the quotient of foreground value (the number of DEGs) and background value (total gene amount). The circle size represents the genes number, the colors indicate the significance factor.

The total number of differentially expressed genes (DEGs) in each treatment is shown in Figure 5A. The genes/transcripts with a false discovery rate (FDR) below 0.05 and absolute fold change (FC) ≥ 2 (or log 2 |FC| ≥ 1) were considered differentially expressed. After CuO-NPs treatment for 15 days, 2270 gene transcripts (1742 upregulated and 528 downregulated) were differentially expressed in the low concentration (100 mg/L CuO-NPs, defined as T1). At the highest concentration (1000 mg/L CuO-NPs, defined as T2), more gene transcripts (4264) were differentially expressed, including 3855 upregulated and 409 downregulated (Figure 5A). Among these were genes related to the pathway of photosynthesis, plant hormone signal transduction, ABC transports, Endocytosis, flavonoid biosynthesis, glutathione metabolism, peroxisome, etc. (Figure 5B). Also, 1201 gene transcripts were differentially expressed between T1 and T2 (Figure 5A). These differential transcripts reveal an exposure concentration-dependent decrease or increase in the transcript levels.

### 2.5. RNA Sequencing Validation by qPCR

To confirm our transcriptome results, quantitative analysis of gene expression was detected using real-time quantitative PCR (qPCR). Nine genes involved in photosynthesis, antioxidant activities, metal transport, and hormone signal transduction were selected randomly from the microarray results. In 15-day CuO-NPs treated lettuce, the expressions of chlorophyll a/b-binding protein 6A (*CAB6A*), photosystem I reaction center subunit psaK (*psaK*), photosystem II reaction center protein W (*psbW*), and light-harvesting chlorophyll a/b-binding protein 5 (*LHCB5*) were significantly decreased in T2 (*p* < 0.05) (Figure S2). Manganese/iron superoxide dismutase (*FSD2*) was upregulated in T1 and T2. ABC transporter C family member 3 (*ABCC3*) was down regulated in T1 and T2. The expression of copper transporter 5.1-like (*COPT5.1*) was significantly decreased both in T1 and T2. Meanwhile, auxin-induced protein 22D (*AUX22D*) was upregulated in T1 and T2. Ethylene response factor 1B (*ERF1B*) was downregulated considerably in T1 (Figure S2).

**Figure 6:**
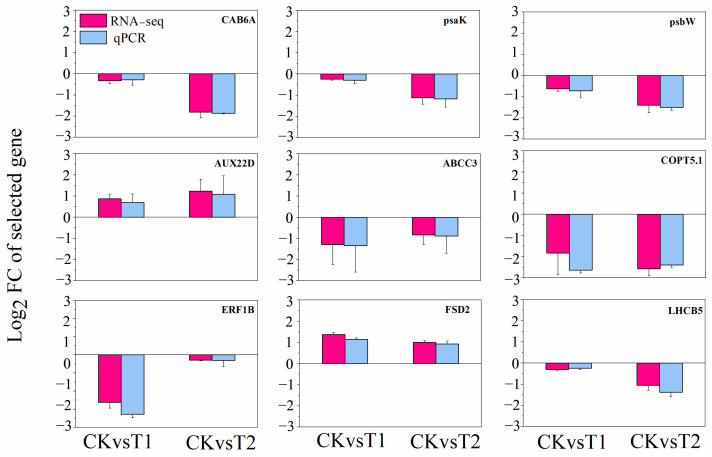
qPCR validations of nine selected DEGs in lettuce after 15 days of foliar exposure to CuO-NPs (0 (CK), 100 (T1), and 1000 (T2) mg/L). The housekeeping gene actin (ACT7) was chosen as the internal reference gene. Values are expressed as the mean of three replicates (± SD) for each treatment.

Compared to the transcriptome data, there was no significant difference between qPCR analysis and RNA sequencing (RNA-Seq), except that the log2 fold-changes (log2 FC) of *ERF1B* gene was slight higher in qPCR results than that in RNA-Seq in T1 treatment (Figure 6). The log2 FC and the variation trends of all the nine selected genes observed in qPCR analysis and RNA-Seq were consistent. This result suggests that the RNA-seq is valid and can be used for subsequent analysis.

### 2.6. Gene Expression Pattern Analysis, Clustering, and Functional Enrichment of DEGs

**Figure 7:**
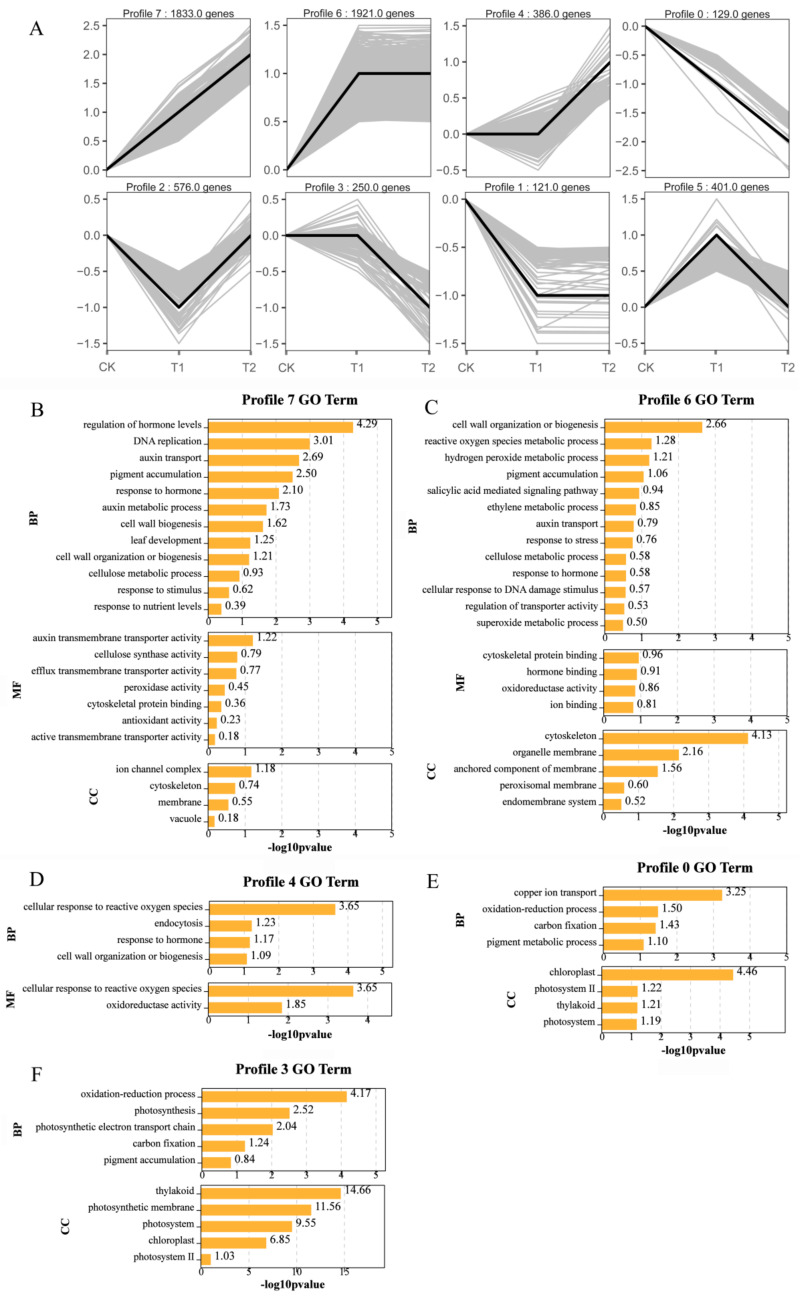
Patterns of gene expressions across three treatments (0 (CK), 100 (T1), and 1000 (T2) mg/L) inferred by STEM analysis (**A**); in each frame, the black line represented the expression tendency of all the genes; the number of genes belonging to each pattern was labeled above the frame. GO enrichment analysis of profile 7 (**B**), profile 6 (**C**), profile 4 (**D**), profile 0 (**E**), and profile 3 (**F**) after 15 days of foliar exposure to CuO-NPs.

The genes displayed a considerable difference in expression profiles in response to CuO-NPs stress between different exposure doses (Figure 7A). The total DEGs at varying exposure concentrations (0 (CK), 100 (T1), and 1000 (T2) mg/L) were clustered into eight profiles (from profile 0 to 7) based on the expression patterns of genes using the Short Time-series Expression Miner (STEM) software (Figure 7A). The most represented clusters are profile 7 and 6 (*p* < 0.05). In profile 7, the expressions of 1833 gene transcripts increased concomitantly with the significant increase of CuO-NP (T1 and T2), and in profile 6, the expression of 1921 gene transcripts was increased in T1 but plateaued at T2. Moreover, 386 gene transcripts showed an increased expression level at T2 (profile 4), while 500 gene transcripts decreased following CuO-NP treatment (profiles 0, 1, and 3). In summary, the profile 7, 6, and 4 represent an upward trend of gene expression pattern. The profile 0 and 3 have showed downward trend of gene expression pattern.

To define the functional annotation of the changes in transcription, GO classifications were implemented for the genes belonging to these profiles.

As shown in Figure 7B and Table S2, the DEGs of profile 7 were assigned into 23 GO terms in all the three GO categories. The enriched biological processes (BP) included regulation of hormone levels, auxin transport, pigment accumulation, hormone response, leaf development, cell wall organization or biogenesis, cellulose metabolism, and response to the stimulus. The overrepresented Gene Ontology (GO) terms related to cellular component (CC) were ion channel complex, cytoskeleton, membrane, and vacuole. The overrepresented GO terms related to molecular function (MF) included auxin transmembrane transporter activity, cellulose synthase activity, efflux transmembrane transporter activity, peroxidase activity, cytoskeletal protein binding, anion binding, and antioxidant activity.

In profile 6 (Figure 7C and Table S2), 22 GO terms were enriched in all the three GO categories. The overrepresented GO terms related to BP included cell wall organization or biogenesis, ROS metabolic processes, H_2_O_2_ metabolic process, pigment accumulation, salicylic acid-mediated signaling, ethylene metabolic processes, auxin transport, stress response, cellulose metabolism, hormone response, and regulation of transmembrane transporter activity. The overrepresented GO terms were related to the CC cytoskeleton, peroxisomal membrane, and endomembrane system. The overrepresented GO terms related to MF included cytoskeletal protein binding, hormone binding, oxidoreductase activity, ion binding, and cellulose synthase activity. The expression level of these genes peaked at T1 and maintained at a high level during the subsequent stage.

The DEGs in profile 4 increased only after T2 treatment, including 386 gene transcripts (Figure 7D and Table S2). The enriched 6 GO terms were related to the cellular response to ROS, endocytosis, hormone responses, cell wall organization or biogenesis, oxidoreductase activity, and transporter activity.

The DEGs in profiles 0 and 3 decreased at T1 and/or T2, 8 and 10 GO terms were enriched in the categories of BP and CC for profile 0 and 3, respectively. The genes involved in copper ion transport, oxidation-reduction processes, photosynthesis (pigment metabolic processes, chloroplast, thylakoid, photosystem, photosynthetic electron transport chain, regulation of photosynthesis, light reaction, pigment accumulation, and photosynthetic membranes), and carbon fixation (profiles 0 and 3) (Figure 7E,F and Table S2).

The results indicate that the expression level of most of genes related to cell wall organization or biogenesis, ROS metabolic processes, auxin transport, hormone response, stress response, regulation of transmembrane transporter activity, transporter activity, cytoskeletal protein binding, and antioxidant activity were increased, but the transcription level of genes involved in photosynthesis was obviously decreased after CuO-NPs exposure.

The transcriptomic responses of plants to metal-NP were also reported in several other studies. In a study on *Arabidopsis thaliana*, Landa et al. (2012) reported that ZnO and TiO_2_-NPs upregulated 660 and 80 genes, while they down regulated 826 and 74 genes, respectively, and all the genes whose expression was altered were associated with stress responsiveness [55]. The transcriptomic analysis in the study of Zhang et al. (2018) showed that 206 genes involved in oxidative stress responses were upregulated in wheat (*Triticum aestivum* L.) under the Cu-NP treatment; the transcription of nearly 260 genes involved in transmembrane transport (nitrate transport and auxin efflux transmembrane transport, metal transporters) changed in the Cu-NP and Cu^2+^ treatments compared to the observations made in control plants root [1]. Tumburu et al. (2015) reported that TiO_2_–NPs affected the expression of genes involved in DNA metabolism, hormone metabolism, tetrapyrrole synthesis, and photosynthesis of *Arabidopsis thaliana* [56]. According to the study of Simon et al. (2013), enhancement in transcripts encoding cell wall and flagella components and decline in expression of genes associated with photosynthesis were found in *Chlamydomonas reinhardtii* after exposure with Ag, TiO_2_, ZnO, and quantum dots [57]. Additionally, according to the GO annotation, DEGs identified after Cu treatment were mostly involved in gene regulation, energy metabolism, transport, cell processes, stress, antioxidant metabolism, and development in the green microalga [58].

In conclusion, plants established defense and detoxification strategy to manage the toxicity induced by NPs, the genes related to stress responses, cellular responses, and metabolic processes were variably expressed after NPs exposure. The transcriptomic findings offer more basic knowledge of the interaction between NPs and plants, which could provide new insights into the molecular mechanisms of metal NP toxicity on plants.

### 2.7. Significant DEGs

The significant genes involved in cell wall organization or biogenesis, photosynthesis, oxidation-reduction process, antioxidant activity, transport, hormone signal transduction were selected and shown in Table 2.

#### 2.7.1. Changed Cell Wall Organization or Biogenesis

CuO-NPs treatment increased the DEGs related to cell wall organization or biogenesis, generally, more than 47 DEGs are involved in cell wall metabolism, some selected important DEGs (log 2 |FC| ≥ 1) are listed in Table 2. These DEGs include cellulose synthase A catalytic subunit 4 (*CESA4*), cellulose synthase-like protein D5 (*CSLD5*), FASCICLIN-like arabinogalactan protein 8 (*FLA8*), Barwin-like endoglucanase (*EXPA1*) (profile 7); xyloglucan endotransglucosylase/hydrolase 7, 8 (*XTH7*, *XTH8*), Barwin-like endoglucanase (*EXPA6*), carbohydrate-binding domain CBM49 (*At1g64390*), cellulose synthase-like protein D3 (*CSLD3*) (profile 6); Barwin-like endoglucanase (*EXPA4*, *EXPA10*) (profile 4) (Table 2). These genes regulate cell wall biogenesis and organization, cellulose synthase activity and metabolism, glucan metabolism, phloem or xylem histogenesis, etc.; and thereby manage to maintain the cell wall integrity and flexibility of lettuce leaves. The general upregulation of genes involved in cell wall organization or biogenesis is not consistent with our reported negative effect of CuO-NP on leaf area. Plant has defense mechanisms strategies to regulate the damage induced by CuO-NP; however, the regulation in gene expression levels and plant phenotypes are not always synchronized. Plant growth could be influenced by several different parameters, the suppression on leaf area probably results from Cu accumulation, Cu adsorption on leaf surface, the decreased net photosynthesis and photosynthetic pigments contents, and the increased oxidative stress in plants [59]. The regulation on cell wall biogenesis may mitigate a part of the damage caused by CuO-NP, but the toxicity is still severe in lettuce, as evidenced from the decreased lettuce weight and leaf area. Further unambiguous assignments could not be made from our recent data.

Cell walls play a central role in plant and microalgal tolerance to metals [58]. For example, more than 80% of Cu was accumulated in the cell wall of lettuce leaves after CuO-NPs exposure for 5, 10, and 15 days [59]. When the charophyte *Nitellopsis obtusa* was exposed to either Cu-NPs or CuSO_4_ for 3 h, the most significant proportion of Cu accumulated in the cell walls [60]. The pectin, cellulose, hemicellulose, and lignin of plant cells can fix and precipitate heavy metals to decrease cell damage [61]. Cu compartmentalization in the cell wall of plants could be one of the mechanisms inhibiting plant Cu diffusion. The increased gene transcription levels related to cell wall organization or biogenesis in the present study may change the cell wall structure and components and influence NP uptake and leaf performance. The transcription of *XTH* genes involved in xyloglucan metabolic processes [44]. Xyloglucan is a regulatory element involved in cell growth and cell wall differentiation [62]. Our results suggest that cell walls may play an essential role in NPs tolerance in lettuce leaves after CuO-NPs exposure by regulating the cell wall structure and composition.

#### 2.7.2. Decreased Photosynthesis

Generally, more than 42 genes involved in photosynthesis, some selected important DEGs (log 2 |FC| ≥ 1) are listed in Table 2. According to the gene expression patterns and GO and Kyoto Encyclopedia of Genes and Genomes (KEGG) analysis, treating lettuce plants with CuO-NPs resulted in the downregulation of genes involved in photosynthesis at T1 and T2. The DEGs *LHCB5*, photosystem I chlorophyll a/b-binding protein 3 (*LHCA3*), *CAB6A*, chlorophyll a/b-binding protein 3C (*CAB3C*), photosystem I reaction center subunit (*psaK*, *psaD* (ncbi_111898767), *psaL*), and photosystem II reaction center protein (*psbB* and *psbZ*) remained unchanged at T1, but were decreased at T2. The genes *psbW*, *psaD* (ncbi_111894261), cytochrome b6/f complex Fe-S subunit (*petC*), photosystem II reaction center protein I (*psbI*), and cytochrome f (*petA*) decreased with the increase of exposure dose (Table 2). The *psaK* gene was down regulated by more than 415 fold after CuO-NPs treatment compared to control.

**Figure 8:**
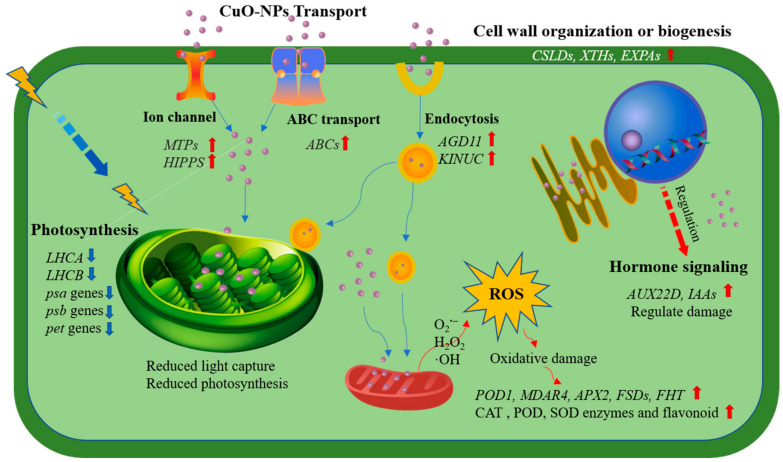
The scheme of lettuce responses to CuO-NPs. The CuO-NPs and Cu^2+^ mainly uptake and transport by ABC family, endocytosis/phagosome (upregulate AGD11, KINUC. etc), HIPPs, MTPs, and metal ion channel; CuO-NPs could have damaging effects on photosynthesis and led to oxidative damage; The increase of antioxidant activity (CAT, POD, and SOD activity and flavonoids content), and the upregulate of cell wall organization or biogenesis genes (CSLDs, XTHs, etc.) and hormone (auxin) level may play an important role in regulating the stresses induced by CuO-NPs.

The results indicate that CuO-NPs treatment downregulated the expressions of the light-harvesting chlorophyll protein complex, photosystem II (*Thermosynechococcus slongatus*), photosystem I (*Thermosynechococcus slongatus*), cytochrome b6/f complex, photosynthetic electron transport, and F-type ATPase (*Escherichia coli*) (Table 2, Figure 8). This finding was consistent with the results of net photosynthesis, chlorophyll content, and chlorophyll fluorescence parameters (F_v_/F_m_, Φ_PSII,_ and ETR) (Figure 2); all decreased significantly after CuO-NPs treatment.

Photosynthesis is a complex process, and most NPs (particularly CuO, Ag, and NiO-NPs) have adverse structural and/or functional effects on it [8,63,64]. The structural effects are mainly due to a decrease in the content of photosynthetic pigments, and to a lesser extent, alterations in grana development and deformation of chloroplasts. At the physiological level, the damage is evidenced through decreased chlorophyll fluorescence, lower photosynthetic efficiency of photosystem II, reduction in ETR and stomatal conductance, and lower net photosynthesis [34]. Previous studies reported that Lhcb1–4, the LHC II encoding genes is crucial in plant photosynthesis. In PSII, the reaction center for LHC II, the substitution of Mg^2+^ in its chlorophyll, is reportedly a target of Cu toxicity [58]. It was discovered that the substitution of magnesium (the central atom of chlorophyll) by heavy metals (mercury, copper, cadmium, nickel, zinc, lead) in vivo is a feasible damage mechanism in stressed plants. This substitution prevents photosynthetic light-harvesting in the affected chlorophyll molecules, resulting in a breakdown of photosynthesis [65]. In the macrophyte *Ceratophyllum demersum*, nanomolar concentrations of Cu affected the PS II reaction center [66]. In the present study, the reduced photochemical reactions can be associated with a decline in chlorophyll contents and oxidative stress due to the 15-days exposure of lettuce plants to CuO-NPs.

#### 2.7.3. Altered Oxidation-Reduction Processes and Antioxidant Activity

CuO-NPs treatment induced the regulation of genes involved in oxidation-reduction processes and antioxidant activity. The selected important DEGs (log 2 |FC| ≥ 1) are listed in Table 2. Generally, the transcriptional level of peroxidase activity protein (*POD1*), monodehydroascorbate reductase 4 (*MDAR4*), and ascorbate peroxidase (*APX2*) were continually upregulated in both T1 and T2 (profile 7). Three genes were upregulated in T1 but plateaued in T2, including manganese/iron superoxide dismutase (*FSD2* and *FSD3*) and flavanone 3-hydroxylase (*FHT*) (profile 6). In both at T1 and T2, the genes encoding iron superoxide dismutase isoform 2 (*SODB*) were downregulated significantly (profile 0), while the transcriptional level of carotenoid oxygenase (*CCD4)* was downregulated in T2 (profile 3) (Table 2).

It is well known that ascorbate peroxidase, superoxide dismutase, and catalase are involved in ROS scavenging and are frequently associated with metal and NP toxicity [27,63,64]. Shen et al. (2010) reported significantly higher transcription levels of the two enzymes (ascorbate peroxidase (APX1) and manganese superoxide dismutase (MSD1)) in leaves of *Arabidopsis* injected with single-walled carbon nanotubes. The expressions of *SOD* and *APX* genes were upregulated in roots of mung bean plants under CuO-NPs exposure (0, 20, 50, 100, 200, and 500 mg/L); the expression of *CAT* gene were upregulated at lower concentrations and reduced under the highest exposure concentrations of CuO-NPs (500 mg/L) [36]. In the study of Nair and Chung (2015), the genes associated with CuZn superoxide dismutase (CuZnSOD) were upregulated, while no significant change was found in expression of *CAT* and *APX* genes [67]. Flavonoids are the largest class of phenolics with antioxidant activities [45,46]. The increased transcription levels of antioxidant enzyme genes (*POD1, MDAR4, APX2, FSD2*, and *FSD3*) and flavanone 3-hydroxylase (*FHT*) gene in the present study indicates the activation of plant’s defense mechanism to counteract the oxidative damage caused as a result of CuO-NPs exposure. The decrease in the expression of *CCD4* gene and carotenoid levels could indicate decreased energy dissipation via the xanthophyll cycle or an increase of oxidative stress, since carotenoids can scavenge ROS [68].

#### 2.7.4. CuO-NPs Transport

The regulation of genes related to CuO-NPs and Cu^2+^ transport were suggested to have contributed to the accumulation and toxicity of CuO-NPs at different doses [1]. As shown in Table 2, we observed the upregulation of a gene belonging to the ATP-binding cassette (ABC) transporter family after CuO-NP treatment. The genes ABC transporter B family member 19 (*ABCB19*), ABC transporter C family member 10 (*ABCC10*), and ABC transporter G family members 5 and 36 (*ABCG5, ABCG36*) were upregulated, and the expression levels increased with the increase of the exposure dose (profile 7). The gene ABC transporter C family member 12 (*ABCC12*) was upregulated at T2 (profile 4) while *ABCC3* was downregulated at T1 (profile 1).

The transcription levels of alpha-tubulin (*TUBA*), beta-tubulin (*TUBB1, TUBB2, TUBB8*), ADP-ribosylation factor GTPase-activating protein AGD11 (*AGD11*), heat shock protein 70 (*Hsc70*), and kinesin motor family protein isoform 1 (*KINUC*) involved in endocytosis were upregulated after CuO-NP treatment (T1 and/or T2).

The heavy metal-associated isoprenylated plant protein (*HIPP*) gene family involved in cation binding and transport (copper transport protein) were increased after CuO-NPs treatment, including *HIPP37*, *HIPP03*, *HIPP01*, *HIPP39*, *HIPP32*, *HIPP07*, *HIPP36*, *HIPP21*, *HIPP31*, *HIPP09*, *HIPP05*, and *HIPP26*.

Moreover, the expression of Cu-transporting ATPase responsive-to-antagonist1 (*RAN1*), involved in cation transmembrane transport, metal ion binding, and cation transport (Cu^2+^ transport), was upregulated with an increase in the exposure dose. The gene monocopper oxidase-like protein SKU5 (*SKU5*) related to cation binding was upregulated in T1 and plateaued at T2. The transcriptional level of metal tolerance protein genes (*MTP4*, *MTP11*), involved in ion transmembrane transporter activity and cation transport, and natural resistance-associated macrophage proteins family metal transporter 6 (*NRAMP6*) were upregulated after CuO-NPs treatment. The expression levels of the gene COPT5.1, which is involved in Cu^2+^ transport, decreased with increased exposure dose levels.

ATP-binding cassette (*ABC*) proteins are powerful transporters that drive the exchange of compounds across various biological membranes, mostly against the existing electrochemical gradients [69]. ABC transporters have been shown to play a central role in vacuolar sequestration, the final detoxification step of potentially toxic chemicals, heavy metals, and metalloids in plants [70]. *AtABCC1*, *AtABCC2*, and *AtABCC3* function as the vacuolar phytochelatin-heavy metal(loid) transporters in *Arabidopsis* [71]. According to Zhang et al. (2018), Cu-NPs and Cu^2+^ upregulated the transcription of heavy metal ABC transporters (*ABCB4*) and downregulated *ABCB11* in wheat [1]. These changes in *ABCB4* and *ABCB11* expressions suggest that wheat plants use ABC transporters to maintain homeostasis under both Cu-NPs and Cu^2+^ treatments, but each with a distinct mechanism [1]. According to Tiwari et al. (2016), the expression of ABC transporter B was suppressed at 25 ppm gold (Au), but its expression was induced at 10 ppm Au [72]. The study by Beauvais-Flück et al. (2019) proposed a possible model of the cellular mechanisms for Cu detoxification and protection in *Elodea nuttallii*: Cu increases intracellular transport, e.g., vesicle trafficking and ABC transport, and induces a flavonoid-mediated detoxification pathway [58].

Recent research has demonstrated the possibility of NPs endocytosis by plant cells [59]. The genes *TUBB8*, *TUBA*, *TUBB2*, and *TUBB1* are related to “cellular processes-transport and catabolism-phagosome”. Xia et al. (2006) reported the uptake of titanium dioxide and Carbon black particles into lose-fitting phagosomes without noticeable mitochondrial damage [73]. Of note, the cellular uptake of nanoparticles mainly includes clathrin-mediated and caveolae-mediated endocytosis, in addition to phagocytosis and macropinocytosis [74]. Auxilin, Hsc70, and synaptojanin are likely involved in the disassembly of the clathrin coat before endocytic vesicles fuse with early endosomes [75].

Metallochaperones, such as HIPPs, facilitate the safe transport of metallic ions inside the cell [76]. HIPPs play essential roles in responses to biotic/abiotic stresses, heavy-metal homeostasis, and detoxification [77,78]. Subcellular localization analysis of cloned HIPPs from *Haynaldia villosa* L. showed that they are expressed on the plasma membrane [77]. The transcription of many HIPPs is altered under heavy metal stresses, indicating that HIPPs may be involved in the homeostasis of these elements [78]. For example, Cd, Hg, Fe, and Cu induced the transcription of *AtHIPP06*, while *AtHIPP26* transcription was induced by Cd and Zn but not Fe or Cu [79,80].

Metal tolerance proteins (MTPs) are crucial for metal transport at the cellular, tissue, and whole plant levels [81]. Fu et al. (2017) observed that several CitMTP genes are significantly upregulated under Cu toxicity [82]. We speculate that the increased transcriptional level of MTPs (*MTP4* and *MTP11*) observed in this study was essential for Cu homeostasis and detoxification in lettuce.

The conserved COPT have been well characterized in *Arabidopsis* [83]. The tonoplast-localized COPT5 functions as a vacuolar copper exporter and facilitates the inter-organ reallocation of copper ions from the root to reproductive organs [84] and is essential for photosynthetic electron transport under Cu limination [85]. In *Arabidopsis*, COPTs restored the growth of a mutant yeast strain under Cu^2+^ treatment, indicating COPTs transport Cu^2+^ [86]. In this study, the decreased expression level of *COPT5.1* may be responsible for the reduced photosynthesis in lettuce leaves exposed to CuO-NPs.

Several other protein families are also involved in Cu detoxification and sequestration, including HMAs [77], cation diffusion facilitators (CDFs) [87], and NRAMPs [88]. In conclusion, the enhanced expressions of the ABC, TUBA, TUBB, AGD11, HSC-2, KINUC, HIPP, and MTP family genes in CuO-NP treated lettuce in this study may have contributed to the acquisition and translocation of CuO-NP. The downregulation of *ABCC3* and *COPT5.1* may regulate vacuolar phytochelatin-Cu transport, copper ions transfer, and the decreased photosynthetic electron transport.

#### 2.7.5. Hormone Signal Transduction

In this study, about 129 genes are involved in hormone signal transduction. CuO-NPs exposure upregulated the expression of some auxin-responsive and auxin-transport genes in a dose-dependent manner, some selected important DEGs (log 2 |FC| ≥ 1) are listed in Table 2. These genes included *AUX22D*, auxin-responsive SAUR protein (*SAUR50*), auxin-responsive protein IAA9 like, IAA27-like (*IAA9*, *IAA27*), basic-leucine zipper domain-containing protein (*ABF2*), and auxin transport genes, like auxin influx carrier protein (*LAX2*) (profile 7). The gene expression level of CheY-like superfamily (*ARR2*, *ARR6*), phosphotransfer (Hpt) domain-containing protein (*AHP1*), catalytic domain-containing protein (*At3g13560*), auxin efflux carrier component 2 (*PIN2*) were increased in T1 and plateaued at T2 (profile 6). Meanwhile, auxin-responsive protein IAA12-like (*IAA12*), and auxin response factors (*ARF3*, *ARF9*) were upregulated in T2 (profile 4) (Table 2). Notably, the *LAX2* gene (ncbi_111881996) was upregulated by more than 319-fold after CuO-NPs exposure compared to control.

Auxins regulate nearly every aspect of plant growth and development [89]. Both metabolic changes of auxin as well as its transport have been shown to be involved in modulation of plant development [90]. Key to the many functions of auxins is the fact that they are transported cell to cell by at least three families of auxin-specific carrier proteins: the ABCB family of carriers, the PIN-formed (PIN) family of auxin efflux carriers, and the auxin resistant 1/like AUX1 (AUX1/LAX) family of influx carriers [90]. Indole-3-acetic acid (IAA) is the most abundant endogenous auxin [70]. Carrier proteins responsible for the subcellular partitioning of auxins can increase or decrease the nuclear auxin signal [91]. Yu et al. (2016) demonstrated that clathrin regulates auxin maxima and hook formation by modulating PIN3 localization and auxin efflux, suggesting a novel mechanism that integrates developmental signals and environmental cues to regulate plant skotomorphogenesis and photomorphogenesis [92]. Remarkably, auxin influx into protoplasts is primarily (75%) mediated by AUX1 [89]. As auxin is involved in plant growth and development, the increased transcriptional level of auxin-responsive and transport genes observed in this study may be a stress response to CuO-NPs exposure. The existence of regulation/detoxification mechanisms allows the survival of lettuce under conditions of 100 and 1000 mg/L CuO-NPs. We speculate that the plant auxins as well as cell wall and antioxidants play positive role in regulating the stress induced by CuO-NPs.

Taken together, our results reveal the interaction mechanism of CuO-NPs and lettuce through an integrated analysis of the morphological, physiological, and transcriptomic data. A schematic model of our results is shown in Figure 8. The transcriptomic findings reveal a high abundance of genes involved in cell wall organization or biogenesis, photosynthesis, oxidation-reduction, metal transport, and hormonal pathways under CuO-NPs stress. The regulation of these genes might be the key to the optimum growth and development of lettuce.

## 3. Materials and Methods

### 3.1. Exposure of Lettuce Leaves to CuO-NPs

Lettuce seeds were surface-sterilized with 4% sodium hypochlorite (NaClO, Sangon Biotech, Shanghai, China) for 5 min, rinsed three times with distilled water, evenly spread on a petri dish covered with medical gauze and cultured to geminate (one week). Then, lettuces were cultivated under hydroponic conditions with half-strength Hoagland’s nutrient solution. The cultivation was done in a controlled chamber with a day/night temperature range of 25 ± 2 °C (16 h)/20 ± 2 °C (8 h), a 65 ± 5% relative humidity, and a light intensity of 425 ± 50 photons µmol/m^2^·s. Then, the leaves of two weeks old plants were exposed to CuO-NPs. The CuO-NPs used for plant exposure were a commercial product (CAS 1317-38-0, Sigma-Aldrich^®^, St. Louis, Missouri, USA). The primary particle size ranged from 40 to 200 nm, with a specific area of 12.6 m^2^/g and 99.9% purity, as documented in our previous study [59]. CuO-NPs suspensions were prepared at 0, 100, and 1000 mg/L, then homogenized by sonication for 30 min in a water bath (at 240 w and 40 KHz, Ultrasonic vibration generator, JP-040, Jiemeng, China). The CuO-NPs were applied as droplets of CuO-NP suspension to the adaxial surface of lettuce leaves by a pipette. A total of 40 µL were deposited on every plant every 12 h. The cultivation containers consisted of pots with lids that had a small hole. Dry sponge mats were placed in the tiny holes to avoid root contact with CuO-NPs, as described by Xiong et al. (2020) [59]. Each treatment was performed in five replicates.

Fifteen days after treatment, leaf and root samples were collected, and their fresh weights were determined. The leaf area of each sample was quantified by Image J 1.46r (National Institutes of Health, Bethesda, MD, USA).

### 3.2. Gas Exchange Parameters, Photosynthetic Pigments Concentration, and Chlorophyll Fluorescence Parameters

Gas exchange parameters, P_N_, g_s_, C_i_, and T_r_ were quantified by a Li6400 Portable Photosynthesis System (Li-COR, Lincoln, NE, USA) using the first fully expanded leaf after 15-day CuO-NPs treatment. The measurements were performed between 9:00 a.m. and 11:00 a.m. under a leaf chamber temperature of 25 °C and a light intensity of 1500 µmol/m^2^·s. Each treatment was performed in five replicates.

Photosynthetic pigments, chlorophyll a, chlorophyll b, total chlorophyll, and carotenoids were extracted from fresh leaves after 15-day treatment. The extractions were done with 80% acetone at 4 ℃ for 24 h in darkness [27]. The pigment quantities were calculated from the absorbance at 663 nm, 645 nm, and 470 nm, measured with a spectrophotometer (Beckman Coulter DU730 U*v*/*v*IS spectrophotometer, USA). Each treatment was performed in five replicates.

The chlorophyll fluorescence parameters were detected by a portable modulating chlorophyll fluorescence instrument PAM-2500 fluorometer (Walz, Effeltrich, Germany) as described by Zhang et al. (2019), with minor modifications [27]. About 20 treated seedlings were dark-adapted for at least 20 min before evaluating chlorophyll fluorescence. The parameters included F_v_/F_m_, Φ_PSII_, ETR, and NPQ.

### 3.3. ROS Determination and Antioxidant Enzyme Activities in Plant Leaves 

Hydrogen peroxide in lettuce leaves was determined by the ROS-sensitive dye 3′3′-diaminobenzidine (DAB) following the methods described by Ma et al. (2015) and Shaw and Hossain (2013) [20,93]. Plant leaves from the control and NP-treated plants were incubated in 0.5 mg mL^−1^ DAB (MYM Biological Technology Company Limited, Beijing, China) in phosphate buffer (50 mM, pH 7.0), vacuumed for four times (15 min each), and incubated at room temperature overnight (about 9 h). The stained leaves were boiled in 95% ethanol (95 °C) for 5 min to remove chlorophyll. After washing with distilled water, tissue images were captured using a Leica DM3000 fluorescence microscope (Leica Microsystems CMS GmbH, Wetzlar, Germany) equipped with a digital camera (Leica DFC7000 T). A deep-brown polymerization product indicated H_2_O_2_ accumulation after reacting with DAB. The H_2_O_2_ generation in plant leaves was quantified by the image processing and analysis software Image J 1.46r (National Institutes of Health, Bethesda, Maryland, USA). Each treatment was performed in three replicates.

The formation of superoxide anion in lettuce leaves was detected by staining with nitroblue tetrazolium (NBT) [40]. Lettuce leaves were immersed in 0.1% NBT (MYM Biological Technology Company Limited, Beijing, China) and 10 mmol ^−^^1^ sodium azide (Beijing Dingguo Biotechnology Company Limited, Beijing, China) in phosphate buffer (pH 6.4), vacuumed for four times (15 min each), and then dark-incubated for more than 2 h. The accumulation of O_2_^−^ was indicated by the dark blue insoluble formazan reacting with NBT. The chlorophyll was removed from leaves as described above, and images were recorded with a digital camera (Leica DFC7000 T). The generation of O_2_^−^ in plant leaves was quantified by the image processing and analysis software Image J 1.46r (National Institutes of Health, Bethesda, Maryland, USA). Three replicates were prepared for each treatment.

For determining the activity of POD (EC 1.11.1.7), CAT (EC 1.11.1.6), and SOD (EC 1.15.1.1), the enzyme extraction solution was prepared from 90 mL of 50 mm PBS buffer (pH 7.8), 0.1 mL of 0.1M EDTA, 0.1 mL of 0.1% (*v*/*v*) Triton X-100, 2 g of 2% (*w*/*v*) polyethylene pyrrolidone (PVP) (Sangon Biotech, Shanghai, China), and topped up to 100 mL. Lettuce leaves (0.1 g) were placed in 2 mL of the enzyme extraction solution, grinded and homogenized with the pre-cooled mortar and pestle, and then centrifuged at 12,000 g for 20 min at 4 °C. The supernatant was collected as the enzyme extract. CAT activity was evaluated by the method of Aebi (1984) [94]. The reaction mixture (2 mL) contained 2.9 mL 30 mM H_2_O_2_ (Guangzhou Chemical Reagent Factory, Guangzhou, China) and 0.1 mL of enzyme extract. The decrease in the absorbance was recorded at 240 nm. One unit of enzyme activity was defined as 1 nmol H_2_O_2_ dissociated·min^−1^. The POD activity was determined following the protocol of Hemeda and Klein (1990) [95]. The reaction mixture for POD analysis comprised of 1.875 mL 50 mm phosphate buffer (pH 7.0), 1 mL 30 mm H_2_O_2_ solution, 0.025 mL guaiacol, and 100 µl enzyme extract. The absorbance of the mixture was recorded at 470 nm for 180 s (recorded every 20 s) at 25 °C. The POD activity was expressed as the amount of guaiacol oxidized per min^−1^ mg^−1^ of protein. Five replicates were prepared for each treatment. The activity of SOD was measured according to the total Superoxide Dismutase assay kit (Hydroxylamine method) (Nanjing Jiancheng Bioengineering Institute). One unit of SOD activity was defined as the amount of enzyme required for 1 mg tissue proteins in 1mL of a reaction mixture SOD inhibition rates to 50% as measured at 560 nm. The activities of SOD were demonstrated with U/g proteins.

To determine the total flavonoid content, lettuce leaves (0.05 g) were homogenized in 2 mL 95% methanol with a pre-cooled mortar and centrifuged at 15493 g for 10 min at 4 °C (SIGMA Laborzentrifugen 3K15, Germany). The supernatant was collected, diluted eight times, and then freshly prepared extraction solution (200 μL 5% NaNO_2_, 300 μL 10% Al_3_Cl_3_, and 1 mL 1M NaOH) (Sangon Biotech, Shanghai, China) was added. The absorbance of the reaction mixture was measured at a wavelength of 510 nm (Beckman Coulter DU730 U*v*/*v*IS spectrophotometer, USA). Five replicates were prepared for each treatment. Subsequently, a standard curve of catechins (25–1000 mol/L) was established to calculate the flavonoid content [96].

### 3.4. RNA Extraction, Library Construction, and Sequencing

Total RNA was extracted from the leaf tissue of frozen lettuce (100 mg) using the TRIzol^®^ reagent kit (Invitrogen, Carlsbad, CA, USA) following the manufacturer’s instructions. RNA quality and quantity were assessed on an Agilent 2100 Bioanalyzer (Agilent Technologies, Palo Alto, CA, USA) and further confirmed by RNase free agarose gel electrophoresis. The RNA from all the three replications was pooled, enriched by Oligo(dT) beads and microanalysis for each treatment was performed in biological triplicates. For the transcriptomic analyses, CuO treatments (0, 100, and 1000 mg/L) were defined as CK, T1, and T2, respectively.

Subsequently, the enriched mRNAs were fragmented using a fragmentation buffer and reverse transcribed into cDNA with random primers. Second-strand cDNA was synthesized by DNA polymerase I, RNase H, dNTPs, and buffer. The cDNA fragments were purified with a QiaQuick PCR extraction kit (Qiagen, Venlo, The Netherlands), end-repaired, poly(A) added, and ligated to Illumina sequencing adapters. The ligation products were size selected by agarose gel electrophoresis, PCR amplified and sequenced using Illumina HiSeq2500 by Gene Denovo Biotechnology Co. (Guangzhou, China).

### 3.5. Bioinformatics Analysis

The raw sequence data of all the samples in this study were uploaded to NCBI (https://www.ncbi.nlm.nih.gov/sra/PRJNA710259, 11 March 2021) with the following accession numbers (PRJNA710259). Raw data were filtered by fastp (version 0.18.0) to obtain high-quality clean reads for further analysis [97]. An index of the reference genome of *Lactuca Sativa* (GCF_002870075.1) was built, and paired-end clean reads were mapped to the reference genome using HISAT2.2.4 with “-rna-strandness RF” and other parameters set as a default.

RNAs differential expression analysis between the three treatments was performed by the DESeq2 software between two different groups (and by edgeR between two samples). The genes/transcripts with FDR below 0.05 and absolute FC ≥ 2 (log 2 |FC| ≥ 1) were considered differentially expressed. To obtain detailed information on the toxicity of CuO-NPs at different exposure doses, the DEGs were clustered in eight profiles based on gene expression patterns using the STEM software. STEM is a software program which is used in analyzing short time series microarray gene expression data. DEGs belonging to the same group (profile) were anticipated to have similar patterns of expression over exposure concentrations.

GO terms were identified for genes with a *p*-value < 0.05 in GO-TermFinder. Firstly, all DEGs were mapped to GO terms in the Gene Ontology database (http://www.geneontology.org/, 10 March 2019), and gene numbers were calculated for each term. The significantly enriched GO terms in DEGs were defined by the hypergeometric test. The KEGG pathway enrichment analysis identified significantly enriched metabolic pathways or signal transduction pathways in DEGs by comparing with the whole genome background. The evaluation criterion was similar to that used in the GO analysis. In-house-developed scripts were used to detect significantly enriched DEGs in the KEGG.

### 3.6. Real-Time Quantitative PCR

Quantitative analysis of gene expression was performed using the StepOnePlus™ Real-Time PCR System (ABI, USA) to confirm our transcriptome results. The genes involved in photosynthesis, antioxidant activities, metal transport, and hormone signal transduction were randomly selected from the microarray results. These included *CAB6A*, *psaK*, *psbW*, and *LHCB5*, which are involved in photosynthesis; *FSD2* which participates in antioxidant activities and stress response; *COPT5.1* and *ABCC3* which are essential for metal transport; *ERF1B* and *AUX22D* which are involved in hormone signal transduction. The primer sequences, including that of the reference gene actin/actin-like conserved site-containing protein (*ACT7*) and sequence accession numbers are available in Table S3. The reference gene was stably expressed independent of exposure to the three concentrations of CuO-NPs. The cDNA was synthesized from 2 μg of total RNA in 20 μL of HiScript^®^ II Q RT SuperMix for qPCR (+ gDNA wiper) (Vazyme) according to the manufacturer’s instructions. For PCR amplification, we used 20 μL of a reaction mixture comprising 10 μL of the dye-containing master mix, 0.8 μL primers, 4 μL cDNA, and 5.2 μL ddH_2_O. For each qPCR reaction, three replicate samples of each treatment, taken from the microarray experiments were analyzed, with three technical replicates of each sample run in parallel (*n* = 9). The qPCR data were normalized using the reference gene and analyzed by the comparative delta-delta Ct (2^-ΔΔCt^) method [27,98].

### 3.7. Statistical Analyses

All the values are expressed as mean ± standard deviation (Origin 2019). One-way ANOVA were performed to test the significant differences between treatments, followed by the Duncan’s multiple range test when *p* < 0.05 was statistically significant (PASW Statistics 18).

## 4. Conclusions

According to the present study, we conclude that: (1) ABC family, endocytosis, HIPPs, MTPs, and metal ion binding proteins and channels play essential roles in CuO-NPs and/or Cu^2+^ accumulation and translocation by plant leaves. (2) Both physiological and transcriptomic results showed that CuO-NPs could have damaging effects on photosynthesis and induce ROS accumulation in lettuce leaves after foliar exposure to CuO-NPs. CuO-NPs treatments decrease the net photosynthesis, photosynthetic pigments content, and change chlorophyll fluorescence parameters, and thus influence the plant performance (decrease the biomass and leaf area). (3) Furthermore, the variation of antioxidant enzymes, flavonoids content, cell wall structure and component, and hormone (auxin) may regulate the stresses induced by CuO-NPs.

## Figures and Tables

**Table 1:** Summary of the sequencing reads and read mapping.

Exposure Concentration (mg/L)	Sample	Total Clean Reads	Unique Mapped (%)	Multiple Mapped (%)	Total Mapped (%)	Q30 (%)	GC (%)
0	CK-1	48,984,912	43,707,254 (89.23%)	2,323,237 (4.74%)	46,030,491 (93.97%)	90.54%	45.85%
	CK-2	56,584,572	46,144,248 (81.55%)	4,497,480 (7.95%)	50,641,728 (89.50%)	90.20%	47.17%
	CK-3	51,003,984	44,795,180 (87.83%)	2,680,293 (5.26%)	47,475,473 (93.08%)	90.26%	45.94%
100	T1–1	84,539,086	75,290,760 (89.06%)	4,295,130 (5.08%)	79,585,890 (94.14%)	95.63%	46.16%
	T1–2	70,293,714	59,781,276 (85.04%)	4,518,475 (6.43%)	64,299,751 (91.47%)	95.99%	47.32%
	T1–3	58,663,934	52,706,698 (89.85%)	2,639,439 (4.50%)	55,346,137 (94.34%)	95.29%	45.87%
1000	T2–1	63,165,908	56,834,597 (89.98%)	2,696,569 (4.27%)	59,531,166 (94.25%)	95.25%	45.42%
	T2–2	75,557,582	68,347,699 (90.46%)	3,315,323 (4.39%)	71,663,022 (94.85%)	95.84%	45.68%
	T2–3	52,794,452	46,679,090 (88.42%)	2,503,238 (4.74%)	49,182,328 (93.16%)	95.05%	45.87%
Average total		62,398,683	54,920,756 (87.94%)	3,274,354 (5.26%)	58,195,110 (93.20%)		

Total clean reads: the number of reads after remove low-quality reads, adaptor sequences, poly A and known non-coding RNAs. Unique mapped (%): uniquely compares the number of reads on the reference genome and the proportion of valid reads. Multiple mapped (%): reads the number of reads on the reference genome in multiple comparisons and the proportion of valid reads. Total mapped (%): the total number of reads that can be mapped to the genome and the proportion of valid reads. Q30 percentage is proportion of nucleotides with quality value larger than 30. GC percentage is proportion of guanidine and cytosine nucleotides among total nucleotides.

**Table 2:** Selected genes involved in cell wall organization or biogenesis, photosynthesis, oxidation-reduction process, antioxidant activity, transport, hormone signal transduction

**Gene ID**	**log2 FC**		**Blast Annotation**	**Blasted Species**
	T1	T2		
**Cell wall organization or biogenesis**				
ncbi_111881311	−	1.47	Cellulose synthase A catalytic subunit 4 (*CESA4*)	*Daucus carota*
ncbi_111907519	2.04	3.09	Cellulose synthase-like protein D5 (*CSLD5*)	*Cynara cardunculus*
ncbi_111878486	1.42	1.95	FASCICLIN-like arabinogalactan protein 8 (*FLA8*)	*Cynara cardunculus*
ncbi_111903865	2.32	3.41	Barwin-like endoglucanase (*EXPA1*)	*Cynara cardunculus*
ncbi_111912962	−	1.11	Xyloglucan endotransglucosylase/hydrolase 7 (*XTH7*)	*Cynara cardunculus*
ncbi_111891696	1.39	1.56	Xyloglucan endotransglucosylase/hydrolase 8 (*XTH8*)	*Daucus carota*
ncbi_111905755	1.62	2.06	Barwin-like endoglucanase (*EXPA6*)	*Cynara cardunculus*
ncbi_111916523	3.60	4.59	Barwin-like endoglucanase (*EXPA6*)	*Cynara cardunculus*
ncbi_111894312	−	1.88	Carbohydrate-binding domain CBM49 (*At1g64390*)	*Cynara cardunculus*
ncbi_111918933	1.57	1.24	Cellulose synthase-like protein D3 (*CSLD3*)	*Ziziphus jujuba*
ncbi_111902216	−	1.86	Barwin-like endoglucanase (*EXPA4*)	*Cynara cardunculus*
ncbi_111903657	−	3.70	Barwin-like endoglucanase (*EXPA4*)	*Cynara cardunculus*
ncbi_111876521	−	4.81	Barwin-like endoglucanase (*EXPA10*)	*Cynara cardunculus*
ncbi_111917522	−	8.02	Barwin-like endoglucanase (*EXPA10*)	*Cynara cardunculus*
**Photosynthesis**				
ncbi_111915071	−	−1.31	Light-harvesting chlorophyll a/b-binding protein 5 (*LHCB5*)	*Citrus sinensis*
ncbi_111914553	−	−1.38	Photosystem I chlorophyll a/b-binding protein 3 (*LHCA3*)	*Brassica rapa*
ncbi_111877237	−	−1.83	Chlorophyll a/b-binding protein 6A (*CAB6A*)	*Cynara cardunculus*
ncbi_111914739	−	−2.38	Chlorophyll a/b-binding protein 3C (*CAB3C*)	*Cynara cardunculus*
MSTRG.16095	−8.70	−13.0	Photosystem I PsaG/PsaK domain-containing protein (*psaK*)	*Cynara cardunculus*
ncbi_111880682	−	−1.14	Photosystem I reaction center subunit psaK (*psaK*)	*Daucus carota*
ncbi_111898767	−	−1.74	Photosystem I reaction center subunit *psaD* (*psaD*)	*Glycine max*
ncbi_111894261	−	−1.01	Photosystem I reaction center subu-nit *psaD* (*psaD*)	*Malus domestica*
ncbi_111918291	−	−1.57	Photosystem I reaction center subunit *psaL* (*psaL*)	*Erythranthe guttata*
MSTRG.8984	−	−1.20	Photosystem II CP47 reaction center protein (*psbB*)	*Arachis duranensis*
MSTRG.12634	−	−1.18	Photosystem II 47 kDa protein (*psbB*)	*Lactuca sativa*
ncbi_3772835	−	−1.17	Photosystem II 47 kDa protein (*psbB*)	*Lactuca sativa*
ncbi_3772836	−	−1.39	Photosystem II reaction center protein Z (*psbZ*)	*Lactuca sativa*
ncbi_111881262	−	−1.43	Photosystem II reaction center protein W (*psbW*)	*Cynara cardunculus*
ncbi_111895461	−	−1.06	Cytochrome b6-f complex Fe-S subunit (*petC*)	*Cynara cardunculus*
MSTRG.22911	−	−5.27	Cytochrome b6-f complex Fe-S subunit (*petC*)	*Cynara cardunculus*
ncbi_3772843	−	−1.06	Photosystem II reaction center protein I (*psbI*)	*Phoenix dactylifera*
ncbi_3772900	−	−1.14	Cytochrome f (*petA*)	*Lactuca sativa*
**Oxidation-reduction process, antioxidant activity**				
ncbi_111914667	1.00	2.00	Peroxidase activity protein (*POD1*)	*Populus tomentosa*
ncbi_111907781	−	1.16	Monodehydroascorbate reductase 4, peroxisome (*MDAR4*)	*Vitis vinifera*
ncbi_111882573	−	4.07	Ascorbate peroxidase (*APX2*)	*Ipomoea trifida*
ncbi_111905866	1.02	1.08	Manganese/iron superoxide dismutase (*FSD2*)	*Cynara cardunculus*
ncbi_111884404	1.11	1.47	Manganese/iron superoxide dismutase (*FSD3*)	*Cynara cardunculus*
ncbi_111897600	1.84	−	Flavanone 3-hydroxylase (*FHT*)	*Erigeron breviscapus*
ncbi_111905867	−	−3.32	Iron superoxide dismutase isoform 2 (*SODB*)	*Solanum lycopersicum*
ncbi_111900015	−	−1.58	Carotenoid oxygenase (*CCD4*)	*Lactuca sativa*
**Transport**				
ncbi_111921599	−	1.90	ABC transporter B family member 19 (*ABCB19*)	*Cynara cardunculus*
ncbi_111890853	−	2.33	ABC transporter C family member 10 (*ABCC10*)	*Populus euphratica*
ncbi_111879294	2.44	4.38	ABC transporter G family members 5 (*ABCG5*)	*Cynara cardunculus*
ncbi_111907936	1.47	2.07	ABC transporter G family member 36 (*ABCG36*)	*Beta vulgaris*
ncbi_111910825	−	1.03	ABC transporter C family member 12 (*ABCC12*)	*Vitis vinifera*
ncbi_111888188	−1.23	−	ABC transporter C family member 3 (*ABCC3*)	*Juglans regia*
ncbi_111885307	4.42	5.35	Alpha-tubulin (*TUBA*)	*Cynara cardunculus*
ncbi_111901730	1.56	1.83	Tubulin beta−1 chain (*TUBB1*)	*Sesamum indicum*
ncbi_111900393	1.92	2.42	Tubulin beta-2 chain(*TUBB2*)	*Nelumbo nucifera*
ncbi_111882692	2.97	3.73	Beta-tubulin (*TUBB8*)	*Cynara cardunculus*
ncbi_111906603	−	1.09	ADP-ribosylation factor GTPase-activating protein AGD11 isoform X1 (*AGD11*)	*Cynara cardunculus*
ncbi_111914610	−	5.29	ADP-ribosylation factor GTPase-activating protein AGD11 isoform X1 (*AGD11*)	*Capsicum annuum*
ncbi_111878707	4.65	5.39	Heat shock protein 70 family (*Hsc70*)	*Corchorus olitorius;*
ncbi_111881479	3.67	4.98	Heat shock cognate 70 kDa protein 2 (*Hsc70*)	*Ricinus communis*
ncbi_111913208	−	1.08	Kinesin motor family protein isoform 1 (*KINUC*)	*Theobroma cacao*
ncbi_111921158	−	2.64	Heavy metal-associated isoprenylated plant protein 37 (*HIPP37*)	*Cephalotus follicularis*
ncbi_111882047	−	4.22	Heavy metal-associated isoprenylated plant protein 37 (*HIPP37*)	*Cephalotus follicularis*
ncbi_111886124	−	5.99	Heavy metal-associated isoprenylated plant protein 3 (*HIPP03*)	*Daucus carota*
ncbi_111894055	3.67	3.92	Heavy metal-associated isoprenylated plant protein (*HIPP01*)	*Cynara cardunculus*
ncbi_111894603	1.18	1.28	Heavy metal-associated isoprenylated plant protein (*HIPP39*)	*Cynara cardunculus*
ncbi_111895123	−	1.05	Heavy metal-associated isoprenylated plant protein (*HIPP32*)	*Cynara cardunculus*
ncbi_111895347	2.12	2.91	Heavy metal-associated isoprenylated plant protein (*HIPP07*)	*Daucus carota*
ncbi_111896151	1.35	1.38	Heavy metal-associated isoprenylated plant protein (*HIPP36*)	*Cynara cardunculus*
ncbi_111900275	1.06	−	Heavy metal-associated isoprenylated plant protein (*HIPP21*)	*Cynara cardunculus*
ncbi_111900793	2.52	3.62	Heavy metal-associated isoprenylated plant protein (*HIPP31*)	*Nicotiana sylvestris*
ncbi_111901657	−	1.44	Copper transport protein ATX1 (*HIPP31*)	*Nelumbo nucifera*
ncbi_111903392	4.70	4.92	Heavy metal-associated isoprenylated plant protein (*HIPP09*)	*Cynara cardunculus*
ncbi_111916369	1.13	1.43	Heavy metal-associated isoprenylated plant protein (*HIPP05*)	*Cynara cardunculus*
ncbi_111919298	1.95	1.74	Heavy metal-associated isoprenylated plant protein (*HIPP26*)	*Cynara cardunculus*
ncbi_111900175	−	1.59	Cu-transporting ATPase responsive-to-antagonist1 (*RAN1*)	*Juglans regia*
ncbi_111913891	1.58	1.08	Monocopper oxidase-like protein SKU5 (*SKU5*)	*Erythranthe guttata*
ncbi_111903866	2.35	2.94	Monocopper oxidase-like protein SKU5 (*SKU5*)	*Erythranthe guttata*
ncbi_111913012	−	2.38	Metal tolerance protein 4-like isoform X2 (*MTP4*)	*Nicotiana attenuata*
ncbi_111888942	−	7.82	Cation efflux protein (*MTP11*)	*Cynara cardunculus*
ncbi_111892195	1.57	2.02	Natural resistance-associated macrophage proteins family metal transporter 6 (*NRAMP6*)	*Chengiopanax sciadophylloides*
ncbi_111893192	−1.68	−2.59	Copper transporter 5.1-like (*COPT5.1*)	*Cynara cardunculus*
**Hormone signal transduction**				
ncbi_111883505	−	1.19	Auxin-induced protein 22D (*AUX22D*)	*Daucus carota*
ncbi_111886094	−	1.41	Auxin responsive SAUR protein (*SAUR50*)	*Cynara cardunculus*
ncbi_111896245	−	1.46	Auxin-responsive protein IAA9 like (*IAA9*)	*Zinnia violacea*
ncbi_111909089	−	1.45	Auxin-responsive protein IAA9 like (*IAA27*)	*Prunus mume*
ncbi_111911429	−	1.03	Basic-leucine zipper domain-containing protein (*ABF2*)	*Cynara cardunculus*
ncbi_111898981	1.35	2.12	Auxin transporter-like protein 2 (*LAX2*)	*Dorcoceras hygrometricum*
ncbi_111881996	8.32	8.87	Auxin influx carrier protein (*LAX2*)	*Zinnia violacea*
ncbi_111901327	−	1.07	Auxin influx carrier protein (*LAX2*)	*Zinnia violacea*
ncbi_111895220	−	2.00	CheY-like superfamily (*ARR2*)	*Cynara cardunculus*
ncbi_111877939	1.98	2.04	CheY-like superfamily (*ARR6*)	*Cynara cardunculus*
ncbi_111921291	1.04	1.06	Signal transduction histidine kinase, phosphotransfer (Hpt) domain-containing protein (*AHP1*)	*Cynara cardunculus*
ncbi_111878847	2.44	2.89	Glycoside hydrolase, catalytic domain-containing protein (*At3g13560*)	*Cynara cardunculus*
ncbi_111902332	−	7.44	Auxin efflux carrier component 2 (*PIN2*)	*Cynara cardunculus*
ncbi_111881242	−	1.20	Auxin-responsive protein IAA12-like (*IAA12*)	*Daucus carota*
ncbi_111894991	1.12	1.74	Auxin response factor 3 isoform X1 (*ARF3*)	*Erythranthe guttata*
ncbi_111898759	−	1.22	Auxin response factor (*ARF9*)	*Cynara cardunculus*

## Data Availability

The raw sequence data of all the samples in this study were uploaded to NCBI (https://www.ncbi.nlm.nih.gov/sra/PRJNA710259, 11 March 2021) with the following accession numbers (PRJNA710259).

## Supplementary Materials

The following are available online at https://www.mdpi.com/article/10.3390/ijms22073688/s1. Figure S1: The chlorophyll a (Chl a) and chlorophyll b (Chl b) content in lettuce leaves after 15 days of foliar exposure to CuO-NPs (0, 100, and 1000 mg/L). Values are expressed as the mean of five replicates (± SD) for each treatment; the different lowercase letters indicate significant difference at *p* < 0.05; Figure S2: The qPCR analysis of nine selected DEGs in lettuce after 15 days of foliar exposure to CuO-NPs (0 (CK), 100 (T1), and 1000 (T2) mg/L). CAB6A, psaK, psbW, and LHCB5 were significantly decreased in T2. FSD2 was upregulated in T1 and T2. ABCC3 was down regulated in T1 and T2. COPT5.1 was significantly decreased both in T1 and T2. AUX22D was upregulated in T1 and T2. ERF1B was downregulated considerably in T1. Values are expressed as the mean of three replicates (± SD) for each treatment; one asterisk and two asterisks indicate values significantly different from the CK (*p* < 0.05 and *p* < 0.01, respectively); “NS” indicates no significant difference compared with the CK, Table S1: Data filtering and quality assessment. RNA Sequencing raw reads were filtered by the fastp software (https://github.com/OpenGene/fastp). Clean reads were generated after remove low-quality reads, adaptor sequences, poly A and known non-coding RNAs from raw data; Table S2: Gene ontology (GO) categories enriched in different profiles (profile 7, 6, 4, 0, 3) in lettuce after 15 days of foliar exposure to CuO-NPs; Table S3: The primer sequences for the nine genes and reference gene used for qPCR verification.

## References

[1] Zhang Z., Ke M., Qu Q., Peijnenburg W.J.G.M., Lu T., Zhang Q., Ye Y., Xu P., Du B., Sun L. (2018). Impact of copper nanoparticles and ionic copper exposure on wheat (*Triticum aestivum* L.) root morphology and antioxidant response. Environ. Pollut..

[2] Rajput V.D., Minkina T., Sushkova S., Mandzhieva S., Fedorenko A., Lysenko V., Bederska-Błaszczyk M., Olchowik J., Tsitsuashvili V., Chaplygin V. (2019). Structural and Ultrastructural Changes in Nanoparticle Exposed Plants. Nanoscience for Sustainable Agriculture.

[3] Rajput V.D., Minkina T., Suskova S., Mandzhieva S., Tsitsuashvili V., Chapligin V., Fedorenko A. (2018). Effects of Copper Nanoparticles (CuO NPs) on Crop Plants: A Mini Review. Bionanoscience.

[4] Soares C., Pereira R., Fidalgo F. (2018). Metal-based nanomaterials and oxidative stress in plants: Current aspects and overview. Phytotoxicity of Nanoparticles.

[5] Tripathi D.K., Shweta, Singh S., Singh S., Pandey R., Singh V.P., Sharma N.C., Prasad S.M., Dubey N.K., Chauhan D.K. (2017). An overview on manufactured nanoparticles in plants: Uptake, translocation, accumulation and phytotoxicity. Plant Physiol. Biochem..

[6] Wu J., Wang G., Vijver M.G., Bosker T., Peijnenburg W.J.G.M. (2020). Foliar versus root exposure of AgNPs to lettuce: Phytotoxicity, antioxidant responses and internal translocation. Environ. Pollut..

[7] Hong J., Peralta-Videa J.R., Rico C., Sahi S., Viveros M.N., Bartonjo J., Zhao L., Gardea-Torresdey J.L. (2014). Evidence of translocation and physiological impacts of foliar applied CeO_2_ nanoparticles on cucumber (*Cucumis sativus*) plants. Environ. Sci. Technol..

[8] Xiong T., Dumat C., Dappe V., Vezin H., Schreck E., Shahid M., Pierart A., Sobanska S. (2017). Copper Oxide Nanoparticle Foliar Uptake, Phytotoxicity, and Consequences for Sustainable Urban Agriculture. Environ. Sci. Technol..

[9] Gogos A., Knauer K., Bucheli T.D. (2012). Nanomaterials in Plant Protection and Fertilization: Current State, Foreseen Applications, and Research Priorities Foreseen Applications, and Research Priorities. J. Agric. Food Chem..

[10] Trujillo-Reyes J., Majumdar S., Botez C.E., Peralta-Videa J.R., Gardea-Torresdey J.L. (2014). Exposure studies of core-shell Fe/Fe_3_O_4_ and Cu/CuO NPs to lettuce (*Lactuca sativa*) plants: Are they a potential physiological and nutritional hazard? J. Hazard. Mater..

[11] Zhao L., Ortiz C., Adeleye A.S., Hu Q., Zhou H., Huang Y., Keller A.A. (2016). Metabolomics to Detect Response of Lettuce (*Lactuca sativa*) to Cu(OH)_2_ Nano-pesticides: Oxidative Stress Response and Detoxification Mechanisms. Environ. Sci. Technol..

[12] Keller A.A., Huang Y., Nelson J. (2018). Detection of nanoparticles in edible plant tissues exposed to nano-copper using sin-gle-particle ICP-MS. J. Nanoparticle Res..

[13] Hussain A., Ali S., Rizwan M., Zia ur Rehman M., Javed M.R., Imran M., Chatha S.A.S., Nazir R. (2018). Zinc oxide nanoparticles alter the wheat physiological response and reduce the cadmium uptake by plants. Environ. Pollut..

[14] Rossi L., Fedenia L.N., Sharifan H., Ma X., Lombardini L. (2019). Effects of foliar application of zinc sulfate and zinc nanoparticles in coffee (*Coffea arabica* L.) plants. Plant Physiol. Biochem..

[15] Rizwan M., Ali S., Qayyum M.F., Ok Y.S., Adrees M., Ibrahim M., Zia-ur-Rehman M., Farid M., Abbas F. (2017). Effect of metal and metal oxide nanoparticles on growth and physiology of globally important food crops: A critical review. J. Hazard. Mater..

[16] Gkanatsiou C., Karamanoli K., Menkissoglu-Spiroudi U., Dendrinou-Samara C. (2019). Composition effect of Cu-based nanoparticles on phytopathogenic bacteria. Antibacterial studies and phytotoxicity evaluation. Polyhedron.

[17] Adhikari T., Sarkar D., Mashayekhi H., Xing B. (2016). Growth and enzymatic activity of maize (*Zea mays* L.) plant: Solution culture test for copper dioxide nano particles. J. Plant Nutr..

[18] Hong J., Rico C.M., Zhao L., Adeleye A.S., Keller A.A., Peralta-Videa J.R., Gardea-Torresdey J.L. (2015). Toxic effects of cop-per-based nanoparticles or compounds to lettuce (*Lactuca sativa*) and alfalfa (*Medicago sativa*). Environ. Sci. Process. Impacts.

[19] Shaw A.K., Ghosh S., Kalaji H.M., Bosa K., Brestic M., Zivcak M., Hossain Z. (2014). Nano-CuO stress induced modulation of antioxidative defense and photosynthetic performance of Syrian barley (*Hordeum vulgare* L.). Environ. Exp. Bot..

[20] Shaw A.K., Hossain Z. (2013). Impact of nano-CuO stress on rice (*Oryza sativa* L.) seedlings. Chemosphere.

[21] Wang S., Liu H., Zhang Y., Xin H. (2015). The effect of CuO NPs on reactive oxygen species and cell cycle gene expression in roots of rice. Environ. Toxicol. Chem..

[22] Wu S.G., Huang L., Head J., Ball M., Tang Y.J., Chen D. (2014). Electrospray Facilitates the Germination of Plant Seeds Elec-trospray Facilitates the Germination of Plant Seeds. Aerosol Air Qual. Res..

[23] Hong J., Wang L., Sun Y., Zhao L., Niu G., Tan W., Rico C.M., Peralta-Videa J.R., Gardea-Torresdey J.L. (2015). Foliar applied nanoscale and microscale CeO_2_ and CuO alter cucumber (*Cucumis sativus*) fruit quality. Sci. Total Environ..

[24] Atha D.H., Wang H., Petersen E.J., Cleveland D., Holbrook R.D., Jaruga P., Dizdaroglu M., Xing B., Nelson B.C. (2012). Copper oxide nanoparticle mediated DNA damage in terrestrial plant models. Environ. Sci. Technol..

[25] Da Costa M.V.J., Sharma P.K. (2016). Effect of copper oxide nanoparticles on growth, morphology, photosynthesis, and antioxidant response in *Oryza sativa*. Photosynthetica.

[26] Zuverza-Mena N., Martínez-Fernández D., Du W., Hernandez-Viezcas J.A., Bonilla-Bird N., López-Moreno M.L., Komárek M., Peralta-Videa J.R., Gardea-Torresdey J.L. (2016). Exposure of engineered nanomaterials to plants: Insights into the physiological and biochemical responses-A review. Plant Physiol. Biochem..

[27] Zhang C.L., Jiang H.S., Gu S.P., Zhou X.H., Lu Z.W., Kang X.H., Yin L., Huang J. (2019). Combination analysis of the physiology and transcriptome provides insights into the mechanism of silver nanoparticles phytotoxicity. Environ. Pollut..

[28] Chen Z., Gao S., Jin M., Sun S., Lu J., Yang P., Bond P.L., Yuan Z., Guo J. (2019). Physiological and transcriptomic analyses reveal CuO nanoparticle inhibition of anabolic and catabolic activities of sulfate-reducing bacterium. Environ. Int..

[29] Wang L., Gong H., Liao W., Wang Z. (2015). Accumulation of particles on the surface of leaves during leaf expansion. Sci. Total Environ..

[30] Yue L., Zhao J., Yu X., Lv K., Wang Z., Xing B. (2018). Interaction of CuO nanoparticles with duckweed (*Lemna minor*. L): Uptake, distribution and ROS production sites. Environ. Pollut..

[31] Agathokleous E., Feng Z.Z., Peñuelas J. (2020). Chlorophyll hormesis: Are chlorophylls major components of stress biology in higher plants?. Sci. Total Environ..

[32] Nekrasova G.F., Ushakova O.S., Ermakov A.E., Uimin M.A., Byzov I. (2011). V Effects of copper(II) ions and copper oxide nano-particles on *Elodea densa* Planch. Russ. J. Ecol..

[33] Burzyński M., Kłobus G. (2004). Changes of photosynthetic parameters in cucumber leaves under Cu, Cd, and Pb stress. Photosynthetica.

[34] Tighe-Neira R., Carmora E., Recio G., Nunes-Nesi A., Reyes-Diaz M., Alberdi M., Rengel Z., Inostroza-Blancheteau C. (2018). Metallic nanoparticles influence the structure and function of the photosynthetic apparatus in plants. Plant Physiol. Biochem..

[35] Lalau C.M., Mohedano R.D.A., Schmidt É.C., Bouzon Z.L., Ouriques L.C., Dos Santos R.W., Da Costa C.H., Vicentini D.S., Matias W.G. (2014). Toxicological effects of copper oxide nanoparticles on the growth rate, photosynthetic pigment content, and cell morphology of the duckweed *Landoltia punctata*. Protoplasma.

[36] Gopalakrishnan Nair P.M., Kim S.H., Chung I.M. (2014). Copper oxide nanoparticle toxicity in mung bean (*Vigna radiata* L.) seedlings: Physiological and molecular level responses of in vitro grown plants. Acta Physiol. Plant..

[37] El-Kassas H.Y., Okbah M.A.E.A. (2017). Phytotoxic effects of seaweed mediated copper nanoparticles against the harmful alga: *Lyngbya majuscula*. J. Genet. Eng. Biotechnol..

[38] Natasha, Shahid M., Farooq A.B.U., Rabbani F., Khalid S., Dumat C. (2020). Risk assessment and biophysiochemical responses of spinach to foliar application of lead oxide nanoparticles: A multivariate analysis. Chemosphere.

[39] Dappe V., Dumez S., Bernard F., Hanoune B., Cuny D., Dumat C., Sobanska S. (2019). The role of epicuticular waxes on foliar metal transfer and phytotoxicity in edible vegetables: Case of *Brassica oleracea* species exposed to manufactured particles. Environ. Sci. Pollut. Res..

[40] Singh A., Singh N.B., Hussain I., Singh H. (2017). Effect of biologically synthesized copper oxide nanoparticles on metabolism and antioxidant activity to the crop plants *Solanum lycopersicum* and *Brassica oleracea* var. botrytis. J. Biotechnol..

[41] Cassana F.F., Falqueto A.R., Braga E.J.B., Peters J.A., Bacarin M.A. (2010). Chlorophyll a fluorescence of sweet potato plants cultivated in vitro and during ex vitro acclimatization. Brazil. J. Plant Physiol..

[42] Perreault F., Oukarroum A., Pirastru L., Sirois L., Gerson Matias W., Popovic R. (2010). Evaluation of Copper Oxide Nanoparticles Toxicity Using Chlorophyll a Fluorescence Imaging in *Lemna gibba*. J. Bot..

[43] Foyer C.H., Noctor G. (2003). Redox sensing and signalling associated with reactive oxygen in chloroplasts, peroxisomes and mitochondria. Physiol. Plant..

[44] Jin Y., Fan X., Li X., Zhang Z., Sun L., Fu Z., Lavoie M., Pan X., Qian H. (2017). Distinct physiological and molecular responses in Arabidopsis thaliana exposed to aluminum oxide nanoparticles and ionic aluminum. Environ. Pollut..

[45] Hernández I., Alegre L., Van Breusegem F., Munné-Bosch S. (2009). How relevant are flavonoids as antioxidants in plants?. Trends Plant Sci..

[46] Gill S.S., Tuteja N. (2010). Reactive oxygen species and antioxidant machinery in abiotic stress tolerance in crop plants. Plant Physiol. Biochem..

[47] Soares C., Carvalho M.E.A., Azevedo R.A., Fidalgo F. (2019). Plants facing oxidative challenges—A little help from the antioxidant networks. Environ. Exp. Bot..

[48] Sharma P., Jha A.B., Dubey R.S., Pessarakli M. (2012). Reactive Oxygen Species, Oxidative Damage, and Antioxidative Defense Mechanism in Plants under Stressful Conditions. J. Bot..

[49] Fryzova R., Pohanka M., Martinkova P., Cihlarova H., Brtnicky M., Hladky J., Kynicky J. (2017). Oxidative Stress and Heavy Metals in Plants. Rev. Environ. Contam. Toxicol..

[50] Bela K., Horváth E., Gallé Á., Szabados L., Tari I., Csiszár J. (2015). Plant glutathione peroxidases: Emerging role of the antioxidant enzymes in plant development and stress responses. J. Plant Physiol..

[51] Jonapá-Hernández F., Gutiérrez-Miceli F., Santos-Espinosa A., Ruíz-Lau N., Ruíz-Valdiviezo V., Valdez-Salas B., Gonzá-lez-Mendoza D. (2020). Foliar application of green nanoparticles in *Annona muricata* L. plants and their effects in physiological and biochemical parameters. Biocatal. Agric. Biotechnol..

[52] Quiterio-Gutiérrez T., Ortega-Ortiz H., Cadenas-Pliego G., Hernández-Fuentes A.D., Sandoval-Rangel A., Be-navides-Mendoza A., Cabrera-De La Fuente M., Juárez-Maldonado A. (2019). The application of selenium and copper nanoparticles modifies the biochemical responses of tomato plants under stress by *Alternaria Solani*. Int. J. Mol. Sci..

[53] Dimkpa C.O., Singh U., Bindraban P.S., Adisa I.O., Elmer W.H., Gardea-torresdey J.L., White J.C. (2019). Addition-omission of zinc, copper, and boron nano and bulk oxide particles demonstrate element and size-specific response of soybean to mi-cronutrients exposure. Sci. Total Environ..

[54] Ogunkunle C.O., Jimoh M.A., Asogwa N.T., Viswanathan K., Vishwakarma V., Fatoba P.O. (2018). Effects of manufactured nano-copper on copper uptake, bioaccumulation and enzyme activities in cowpea grown on soil substrate. Ecotoxicol. Environ. Saf..

[55] Landa P., Vankova R., Andrlova J., Hodek J., Marsik P., Storchova H., White J.C., Vanek T. (2012). Nanoparticle-specific changes in *Arabidopsis thaliana* gene expression after exposure to ZnO, TiO_2_, and fullerene soot. J. Hazard. Mater..

[56] Tumburu L., Andersen C.P., Rygiewicz P.T., Reichman J.R. (2015). Phenotypic and genomic responses to titanium dioxide and cerium oxide nanoparticles in *Arabidopsis germinants*. Environ. Toxicol. Chem..

[57] Simon D.F., Domingos R.F., Hauser C., Hutchins C.M., Zerges W., Wilkinson K.J. (2013). Transcriptome sequencing (RNA-seq) analysis of the effects of metal nanoparticle exposure on the transcriptome of *Chlamydomonas reinhardtii*. Appl. Environ. Microbiol..

[58] Beauvais-Flück R., Slaveykova V.I., Cosio C. (2019). Comparative study of Cu uptake and early transcriptome responses in the green microalga *Chlamydomonas reinhardtii* and the macrophyte *Elodea nuttallii*. Environ. Pollut..

[59] Xiong T., Zhang T., Xian Y., Kang Z., Zhang S., Dumat C., Shahid M., Li S. (2020). Foliar uptake, biotransformation, and impact of CuO nanoparticles in *Lactuca sativa* L. var. ramosa Hort. Environ. Geochem. Health.

[60] Manusadžianas L., Gylytė B., Grigutytė R., Karitonas R., Sadauskas K., Vitkus R., Šiliauskas L., Vaičiūnienė J. (2017). Accumulation of copper in the cell compartments of charophyte *Nitellopsis obtusa* after its exposure to copper oxide nanoparticle suspension. Environ. Sci. Pollut. Res..

[61] Wu X., Song H., Guan C., Zhang Z. (2020). Boron alleviates cadmium toxicity in Brassica napus by promoting the chelation of cadmium onto the root cell wall components. Sci. Total Environ..

[62] Maris A., Kaewthai N., Eklöf J.M., Miller J.G., Brumer H., Fry S.C., Verbelen J.P., Vissenberg K. (2011). Differences in enzymic properties of five recombinant xyloglucan endotransglucosylase/hydrolase (XTH) proteins of *Arabidopsis thaliana*. J. Exp. Bot..

[63] Soares C., Branco-Neves S., De Sousa A., Azenha M., Cunha A., Pereira R., Fidalgo F. (2018). SiO_2_ nanomaterial as a tool to improve *Hordeum vulgare* L. tolerance to nano-NiO stress. Sci. Total Environ..

[64] Pinto M., Soares C., Pinto A.S., Fidalgo F. (2019). Phytotoxic effects of bulk and nano-sized Ni on *Lycium barbarum* L. grown in vitro–Oxidative damage and antioxidant response. Chemosphere.

[65] Küpper H., Küpper F., Spiller M. (1996). Environmental relevance of heavy metal-substituted chlorophylls using the example of water plants. J. Exp. Bot..

[66] Thomas G., Stärk H.-J., Wellenreuther G., Dickinson B.C., Küpper H. (2013). Effects of nanomolar copper on water plants—Comparison of biochemical and biophysical mechanisms of deficiency and sublethal toxicity under environmentally relevant conditions. Aquat. Toxicol..

[67] Bashri G., Parihar P., Singh R., Patel A., Prasad S.M. (2018). Plant and Nanoparticle Interface at the Molecular Level: An Integrated Overview. Nanomaterials in Plants, Algae, and Microorganisms.

[68] Jahns P., Holzwarth A.R. (2012). The role of the xanthophyll cycle and of lutein in photoprotection of photosystem II. Biochim. Biophys. Acta Bioenerg..

[69] Wilkens S. (2015). Structure and mechanism of ABC transporters. F1000Prime Rep..

[70] Hwang J.U., Song W.Y., Hong D., Ko D., Yamaoka Y., Jang S., Yim S., Lee E., Khare D., Kim K. (2016). Plant ABC Transporters Enable Many Unique Aspects of a Terrestrial Plant’s Lifestyle. Mol. Plant.

[71] Brunetti P., Zanella L., De Paolis A., Di Litta D., Cecchetti V., Falasca G., Barbieri M., Altamura M.M., Costantino P., Cardarelli M. (2015). Cadmium-inducible expression of the ABC-type transporter AtABCC3 increases phytochelatin-mediated cadmium tolerance in *Arabidopsis*. J. Exp. Bot..

[72] Tiwari M., Krishnamurthy S., Shukla D., Kiiskila J., Jain A. (2016). Comparative transcriptome and proteome analysis to reveal the biosynthesis of gold nanoparticles in *Arabidopsis*. Sci. Rep..

[73] Xia T., Kovochich M., Brant J., Hotze M., Sempf J., Oberley T., Sioutas C., Yeh J.I., Wiesner M.R., Nel A.E. (2006). Comparison of the abilities of ambient and manufactured nanoparticles to induce cellular toxicity according to an oxidative stress paradigm. Nano Lett..

[74] Iversen T.G., Skotland T., Sandvig K. (2011). Endocytosis and intracellular transport of nanoparticles: Present knowledge and need for future studies. Nano Today.

[75] Samaj J., Baluska F., Voigt B., Schlicht M., Volkmann D., Menzel D. (2004). Endocytosis, actin cytoskeleton, and signaling. Plant Physiol..

[76] Khan I.U., Rono J.K., Zhang B.Q., Liu X.S., Wang M.Q., Wang L.L., Wu X.C., Chen X., Cao H.W., Yang Z.M. (2019). Identification of novel rice (*Oryza sativa*) HPP and HIPP genes tolerant to heavy metal toxicity. Ecotoxicol. Environ. Saf..

[77] Zhang H., Zhang X., Liu J., Niu Y., Chen Y., Hao Y., Zhao J., Sun L., Wang H., Xiao J. (2020). Characterization of the Heavy-Metal-Associated Isoprenylated Plant Protein (HIPP) Gene Family from *Triticeae* Species. Int. J. Mol. Sci..

[78] De Abreu-Neto J.B., Turchetto-Zolet A.C., De Oliveira L.F.V., Bodanese Zanettini M.H., Margis-Pinheiro M. (2013). Heavy met-al-associated isoprenylated plant protein (HIPP): Characterization of a family of proteins exclusive to plants. FEBS J..

[79] Gao W., Xiao S., Li H.Y., Tsao S.W., Chye M.L. (2009). Arabidopsis thaliana acyl-CoA-binding protein ACBP2 interacts with heavy-metal-binding farnesylated protein AtFP6. New Phytol..

[80] Suzuki N., Yamaguchi Y., Koizumi N., Sano H. (2002). Functional characterization of a heavy metal binding protein Cdl19 from *Arabidopsis*. Plant J..

[81] Shirazi Z., Abedi A., Kordrostami M., Burritt D.J., Hossain M.A. (2019). Genome-wide identification and characterization of the metal tolerance protein (MTP) family in grape (*Vitis vinifera* L.). Biotech.

[82] Fu X.Z., Tong Y.H., Zhou X., Ling L.L., Chun C.P., Cao L., Zeng M., Peng L.Z. (2017). Genome-wide identification of sweet orange (*Citrus sinensis*) metal tolerance proteins and analysis of their expression patterns under zinc, manganese, copper, and cadmium toxicity. Gene.

[83] Dai J., Wang N., Xiong H., Qiu W., Nakanishi H., Kobayashi T., Nishizawa N.K., Zuo Y. (2018). The yellow stripe-like (YSL) gene functions in internal copper transport in peanut. Genes.

[84] Klaumann S., Nickolaus S.D., Fürst S.H., Starck S., Schneider S., Ekkehard Neuhaus H., Trentmann O. (2011). The tonoplast copper transporter COPT5 acts as an exporter and is required for interorgan allocation of copper in *Arabidopsis thaliana*. New Phytol..

[85] Garcia-Molina A., Andrés-Colás N., Perea-García A., Del Valle-Tascõn S., Peñarrubia L., Puig S. (2011). The intracellular arabidopsis COPT5 transport protein is required for photosynthetic electron transport under severe copper deficiency. Plant J..

[86] Sancenón V., Puig S., Mira H., Thiele D.J., Peñarrubia L. (2003). Identification of a copper transporter family in *Arabidopsis* thaliana. Plant Mol. Biol..

[87] Gustin J.L., Loureiro M.E., Kim D., Na G., Tikhonova M., Salt D.E. (2009). MTP1-dependent Zn sequestration into shoot vacuoles suggests dual roles in Zn tolerance and accumulation in Zn-hyperaccumulating plants. Plant J..

[88] Lanquar V., Ramos M.S., Lelièvre F., Barbier-Brygoo H., Krieger-Liszkay A., Krämer U., Thomine S. (2010). Export of vacuolar manganese by AtNRAMP3 and AtNRAMP4 is required for optimal photosynthesis and growth under manganese deficiency. Plant Physiol..

[89] Rutschow H.L., Baskin T.I., Kramer E.M. (2014). The carrier AUXIN RESISTANT (AUX1) dominates auxin flux into *Arabidopsis protoplasts*. New Phytol..

[90] Zazímalová E., Murphy A.S., Yang H., Hoyerová K., Hosek P. (2010). Auxin transporters--why so many?. Cold Spring Harb. Perspect. Biol..

[91] Li C., Li J., Chong K., Harter K., Lee Y., Leung J., Martinoia E., Matsuoka M., Offringa R., Qu L. (2016). Toward a Molecular Understanding of Plant Hormone Actions. Mol. Plant.

[92] Yu Q., Zhang Y., Wang J., Yan X., Wang C., Xu J., Pan J. (2016). Clathrin-Mediated Auxin Efflux and Maxima Regulate Hypocotyl Hook Formation and Light-Stimulated Hook Opening in Arabidopsis. Mol. Plant.

[93] Ma Y., Zhang P., Zhang Z., He X., Li Y., Zhang J., Zheng L., Chu S. (2015). Origin of the different phytotoxicity and biotransformation of cerium and lanthanum oxide nanoparticles in cucumber. Nanotoxicology.

[94] Aebi H. (1984). Catalase in vitro. Methods in Enzymology.

[95] Hemeda H.M., Klein B.P. (1990). Effects of Naturally Occurring Antioxidants on Peroxidase Activity of Vegetable Extracts. J. Food Sci..

[96] Mishra A., Patel M.K., Jha B. (2015). Non-targeted metabolomics and scavenging activity of reactive oxygen species reveal the potential of *Salicornia brachiata* as a functional food. J. Funct. Foods.

[97] Chen S., Zhou Y., Chen Y., Gu J. (2018). Fastp: An ultra-fast all-in-one FASTQ preprocessor. Bioinformatics.

[98] Zhang S.F., Zhang K., Cheng H.M., Lin L., Wang D.Z. (2020). Comparative transcriptomics reveals colony formation mechanism of a harmful algal bloom species *Phaeocystis globosa*. Sci. Total Environ..

