# Supplementary material for: Dose-Dependent Physiological and Transcriptomic Responses of Lettuce (Lactuca sativa L.) to Copper Oxide Nanoparticles—Insights into the Phytotoxicity Mechanisms
Source: Int J Mol Sci. 2021 Apr 1;22(7):3688. doi: 10.3390/ijms22073688 (PMC8036535; doi:10.3390/ijms22073688)
Supplement: Supplementary file 1 [file ijms-22-03688-s001.pdf]

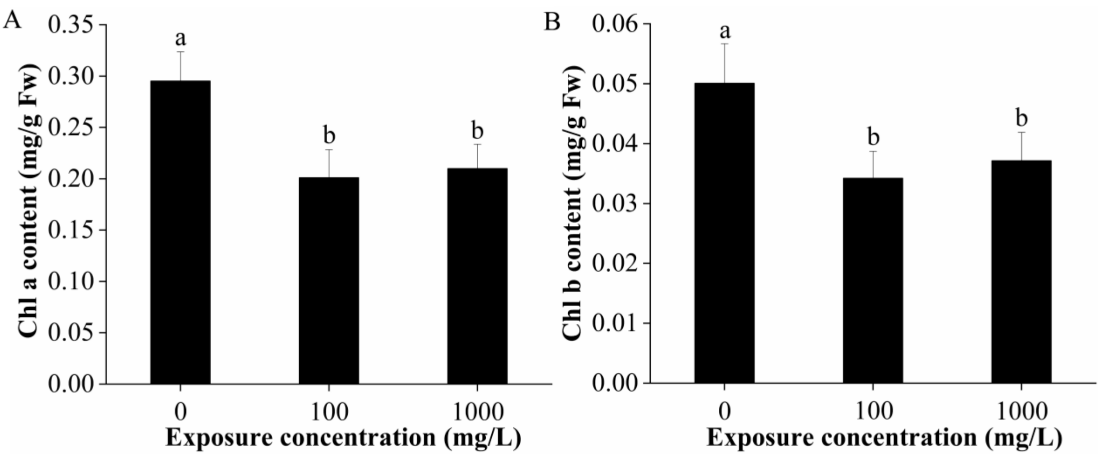

Figure S1. The chlorophyll a (Chl a) and chlorophyll b (Chl b) content in lettuce leaves after 15 days of foliar exposure to CuO-NPs (0, 100, and 1000 mg/L). Values are expressed as the mean of five replicates (± SD) for each treatment; the different lowercase letters indicate significant difference at $p<0.05$.

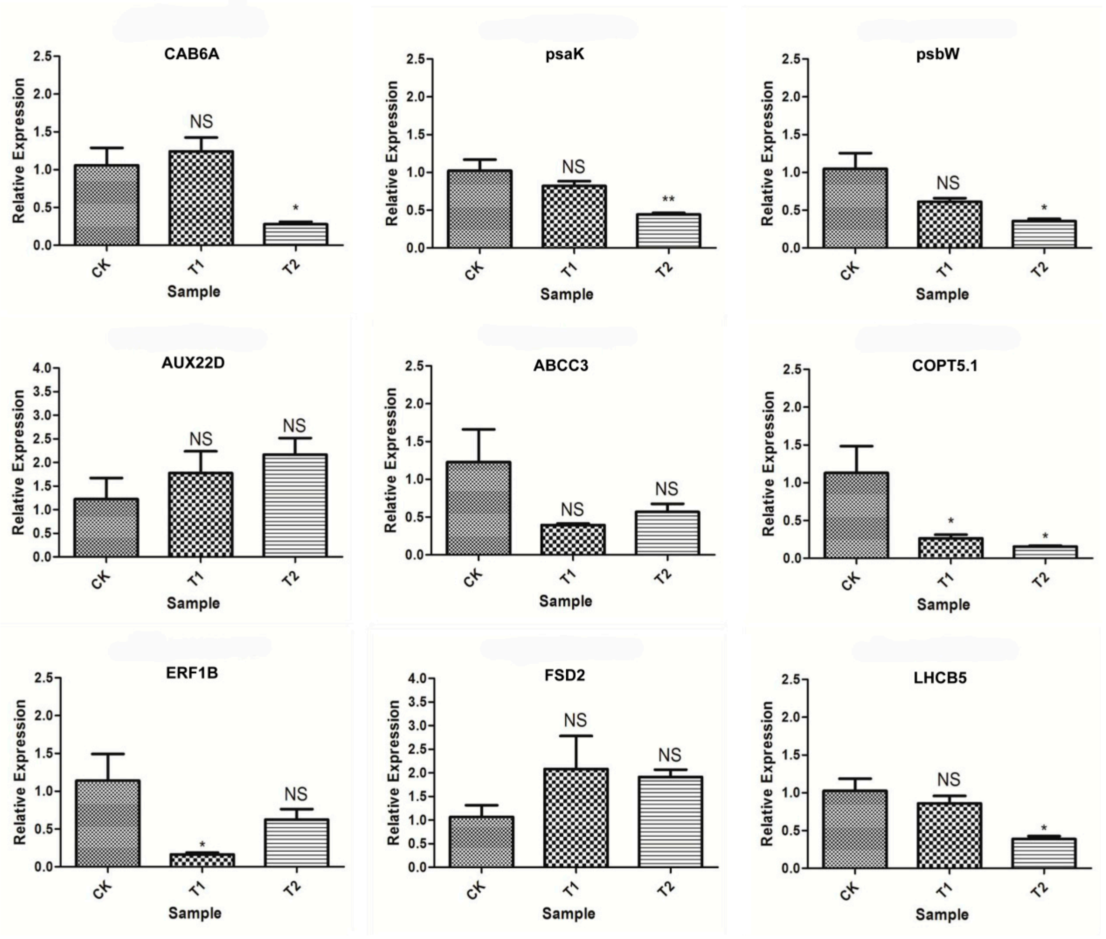

Figure S2. The qPCR analysis of nine selected DEGs in lettuce after 15 days of foliar exposure to CuO-NPs (0 (CK), 100 (T1), and 1000 (T2) mg/L). CAB6A, psaK, psbW, and LHCB5 were significantly decreased in T2. FSD2 was up-regulated in T1 and T2.

ABCC3 was down regulated in T1 and T2. COPT5.1 was significantly decreased both in T1 and T2. AUX22D was up-regulated in T1 and T2. ERF1B was down-regulated considerably in T1. Values are expressed as the mean of three replicates (±SD) for each treatment; One asterisk and two asterisks indicate values significantly different from the CK ($p<0.05$ and $p<0.01$, respectively); "NS" indicates no significant difference compared with the CK.

Table S1. Data filtering and quality assessment. RNA Sequencing raw reads were filtered by the fastp software (https://github.com/OpenGene/fastp). Clean reads were generated after remove low-quality reads, adaptor sequences, poly A and known non-coding RNAs from raw data.

Exposure concentration (mg/L)	Sample	RawDatas	CleanData(%)	Adapter(%)	LowQuality(%)	polyA(%)	N(%)
0	CK-1	49,078,068	48,984,912 (99.81%)	20232 (0.04%)	72920 (0.15%)	0 (0.00%)	4 (0.00%)
	CK-2	56,705,764	56,584,572 (99.79%)	21132 (0.04%)	100060 (0.18%)	0 (0.00%)	0 (0.00%)
	CK-3	51,110,420	51,003,984 (99.79%)	24836 (0.05%)	81596 (0.16%)	0 (0.00%)	4 (0.00%)
100	T1-1	84,694,460	84,539,086 (99.82%)	40018 (0.05%)	109510 (0.13%)	0 (0.00%)	5846 (0.01%)
	T1-2	70,404,864	70,293,714 (99.84%)	30150 (0.04%)	76254 (0.11%)	0 (0.00%)	4746 (0.01%)
	T1-3	58,782,090	58,663,934 (99.80%)	30150 (0.05%)	84066 (0.14%)	0 (0.00%)	3940 (0.01%)
1000	T2-1	63,295,326	63,165,908 (99.80%)	31306 (0.05%)	93822 (0.15%)	0 (0.00%)	4290 (0.01%)
	T2-2	75,667,858	75,557,582 (99.85%)	30104 (0.04%)	75096 (0.10%)	0 (0.00%)	5076 (0.01%)
	T2-3	52,903,148	52,794,452 (99.79%)	25664 (0.05%)	79318 (0.15%)	0 (0.00%)	3714 (0.01%)

Table S2. Gene ontology (GO) categories enriched in different profiles (profile 7, 6, 4, 0, 3) in lettuce after 15 days of foliar exposure to CuO-NPs.

GO ID	Ontology	Description	Gene Ratio	Bg Ratio	*p*value	*p*.adjust
**Profile 7**						
GO:0010817	BP	regulation of hormone levels	10	34	5.10E-05	0.004760253
GO:0006260	BP	DNA replication	7	25	0.000981019	0.05629541
GO:0060918	BP	auxin transport	6	21	0.002030676	0.086160668
GO:0043476	BP	pigment accumulation	5	16	0.003155273	0.091884575
GO:0009725	BP	response to hormone	18	145	0.008031301	0.122879696
GO:0009850	BP	auxin metabolic process	3	9	0.018717469	0.198532134
GO:0042546	BP	cell wall biogenesis	4	17	0.023828197	0.220575587
GO:0048366	BP	leaf development	2	6	0.056549141	0.337443438
GO:0071554	BP	cell wall organization or biogenesis	12	109	0.061123899	0.337443438
GO:0030243	BP	cellulose metabolic process	5	39	0.118342824	0.441025811
GO:0050896	BP	response to stimulus	65	888	0.240607279	0.591241017
GO:0031667	BP	response to nutrient levels	3	34	0.403721656	0.756724511
GO:0080161	MF	auxin transmembrane transporter activity	1	1	0.060442849	0.382974298
GO:0016759	MF	cellulose synthase activity	2	12	0.161293182	0.588643456
GO:0015562	MF	efflux transmembrane transporter activity	1	3	0.170621292	0.588643456

GO:0004601	MF	peroxidase activity	1	7	0.353832256	0.755085329
GO:0008092	MF	cytoskeletal protein binding	3	40	0.43866669	0.787663682
GO:0016209	MF	antioxidant activity	1	14	0.582730417	0.874095625
GO:0022804	MF	active transmembrane transporter activity	6	109	0.653190999	0.895201586
GO:0034702	CC	ion channel complex	1	1	0.066255278	0.468203962
GO:0005856	CC	cytoskeleton	9	96	0.182386401	0.679656307
GO:0016020	CC	membrane	97	1396	0.279459356	0.854936727
GO:0005773	CC	vacuole	1	16	0.666999138	1
**Profile 6**						
GO:0071554	BP	cell wall organization or biogenesis	16	109	0.002199881	0.042861076
GO:0072593	BP	reactive oxygen species metabolic process	9	73	0.053021941	0.33765776
GO:0042743	BP	hydrogen peroxide metabolic process	7	53	0.061177942	0.33765776
GO:0043476	BP	pigment accumulation	3	16	0.086921847	0.380966039
GO:0009863	BP	salicylic acid mediated signaling pathway	3	18	0.114945946	0.466473758
GO:0009692	BP	ethylene metabolic process	2	10	0.140818091	0.484338458
GO:0060918	BP	auxin transport	3	21	0.162225315	0.535704358
GO:0006950	BP	response to stress	35	445	0.173273638	0.553753669
GO:0030243	BP	cellulose metabolic process	4	39	0.262617372	0.660543729
GO:0009725	BP	response to hormone	12	145	0.262667324	0.660543729
GO:0006974	BP	cellular response to DNA damage stimulus	3	27	0.269040425	0.667812317

GO:0032409	BP	regulation of transporter activity	1	5	0.292796574	0.679239932
GO:0006801	BP	superoxide metabolic process	2	17	0.316380034	0.692622931
GO:0008092	MF	cytoskeletal protein binding	5	40	0.110726863	0.671779793
GO:0042562	MF	hormone binding	1	2	0.124352104	0.671779793
GO:0016491	MF	oxidoreductase activity	51	685	0.138374712	0.671779793
GO:0043167	MF	ion binding	70	974	0.156232023	0.671779793
GO:0005856	CC	cytoskeleton	18	96	7.36E-05	0.001633089
GO:0031090	CC	organelle membrane	31	286	0.006905569	0.091896461
GO:0031225	CC	anchored component of membrane	3	10	0.02758272	0.255140156
GO:0005778	CC	peroxisomal membrane	1	4	0.250457116	0.60436391
GO:0012505	CC	endomembrane system	4	40	0.301309474	0.681055622
**Profile 4**						
GO:0034614	BP	cellular response to reactive oxygen species	2	2	0.000225388	0.042147548
GO:0006897	BP	endocytosis	1	4	0.059100458	0.340054944
GO:0009725	BP	response to hormone	5	145	0.067234197	0.380993781
GO:0071554	BP	cell wall organization or biogenesis	4	109	0.081820412	0.417117441
GO:0016491	MF	oxidoreductase activity	16	685	0.014018261	0.162962288
GO:0005215	MF	transporter activity	6	331	0.268374503	0.542583235
**Profile 0**						
GO:0006825	BP	copper ion transport	2	6	0.000564156	0.195197867

GO:0055114	BP	oxidation-reduction process	2	45	0.031978471	0.464349164
GO:0051552	BP	flavone metabolic process	1	6	0.037107992	0.464349164
GO:0042440	BP	pigment metabolic process	1	13	0.078715986	0.464349164
GO:0009507	CC	chloroplast	7	128	3.49E-05	0.000512045
GO:0009523	CC	photosystem II	1	8	0.060750163	0.150994496
GO:0009579	CC	thylakoid	3	118	0.061826432	0.150994496
GO:0009521	CC	photosystem	2	54	0.065167614	0.150994496
**Profile 3**						
GO:0055114	BP	oxidation-reduction process	5	45	6.74E-05	0.006357857
GO:0015979	BP	photosynthesis	3	30	0.002994998	0.169516886
GO:0009767	BP	photosynthetic electron transport chain	2	15	0.009131504	0.30703728
GO:0015977	BP	carbon fixation	1	6	0.057471947	0.451793363
GO:0043476	BP	pigment accumulation	1	16	0.146145861	0.559417575
GO:0009579	CC	thylakoid	17	118	2.18E-15	1.05E-13
GO:0034357	CC	photosynthetic membrane	12	64	2.76E-12	4.42E-11
GO:0009521	CC	photosystem	10	54	2.81E-10	3.37E-09
GO:0009507	CC	chloroplast	11	128	1.42E-07	9.74E-07
GO:0009523	CC	photosystem II	1	8	0.092288054	0.158208093

Table S3. The primer sequences for the nine genes and reference gene used for qPCR verification.

Genes		5'--3'	Tm	Product
COPT5.1	F	TTTGCCGCCTTCTACCAGTT	60	247
	R	CCAGCCCAACGACTATTACCA	60.2	
ERF1B	F	CCCGACTACCGAATACTCACC	58.7	88
	R	GTCGTTGAGATTGAAGGGGAGT	59.8	
AUX22D	F	CCAAAGGCAGTTCTCAACACAA	60.3	347
	R	AACATCACCAACAAGCATCCA	58.5	
FSD	F	AGTTTGGTTCTGGTTGGGCTT	60.3	329
	R	CGCATCTATTTCTGCCATCTCC	61.5	
CAB6A	F	GGCGGGCAAGCAACATAC	59.5	176
	R	GGGTCTTTTGAGTAGCCTAATGG	59.4	
psaK	F	GGCACCATCAGCAAACAGGA	61.5	99
	R	TCAGCAAGAGTGAAGCCAGC	58.7	
psbW	F	TCTCCGCTTCCACTCTCACA	58.7	240
	R	CCGTGCTCATTCTCTCATCCA	60.8	
LHCB5	F	CGGTGTGTCGGAAATGCTTG	62	86
	R	TAAAGGTGGCAGGACTGGAAG	59.4	
ABCC3	F	TTCAGCAAGAGAAATGGCAC	56.1	351
	R	AGTGTCCAAACTACCCATCCA	56.8	
ACT7	F	GAAATCACTGCCCTTGCCC	60.1	169
	R	TGTGAACAATAGATGGACCCGA	60.3	
